# Remodeling Highly Fluorinated Electrolyte via Shielding Agent Regulation toward Practical Lithium Metal Batteries

**DOI:** 10.1002/advs.202404248

**Published:** 2024-10-10

**Authors:** Yutong Yang, Shunchao Ma, Hongxing Yin, Yanan Li, Silin Chen, Yu Zhang, Dan Li, Feilong Dong, Yue Zhang, Haiming Xie, Lina Cong

**Affiliations:** ^1^ National & Local United Engineering Laboratory for Power Battery Department of Chemistry Northeast Normal University Changchun 130022 China; ^2^ State Key Laboratory of Electroanalytical Chemistry Changchun Institute of Applied Chemistry Chinese Academy of Sciences Changchun 130022 China; ^3^ School of Engineering, Faculty of Applied Science University of British Columbia Kelowna BC V1V 1V7 Canada

**Keywords:** fluorinated amide, highly fluorinated electrolyte, practical lithium metal batteries, solvation structure, ultrahigh energy density

## Abstract

Highly fluorinated electrolytes have proved effective in improving electrochemical stability of lithium metal batteries. However, excessive fluorination not only detrimentally impacts the electrolyte ionic conductivity, but also inevitably forms the over‐fluorinated interphases with sluggish ion diffusivity. Herein, a strategy on remodeling Li^+^ solvation structure in highly fluorinated electrolyte aided is proposed by fluorinated amide (FDMA), which denoted as “shielding agent”. Benefitting from FDMA's high donor number (DN) value (22.1), the Li^+^‐dipole (fluoroethylene carbonate (FEC) or trans‐4,5‐Difluoroethylenecarbonate (DFEC)) interaction is interrupted and the participation of FDMA in primary solvation sheath fructify the solid‐electrolyte interphase without scarifying the privilege of fluorinated electrolyte on interphase chemistry. Eventually, the optimal high‐fluorinated electrolyte (FDMA/DFEC + 1.0 mol L^−1^ LiTFSI) with this unique shielding effect displays high ionic conductivity and rapid Li^+^ desolvation behavior, enabling Li||LiNi_0.6_Co_0.2_Mn_0.2_O_2_ (Li||NCM622) to achieve an ultralong cycle‐life of 2000 cycles at 1C with 84.7% capacity retention. Even under extreme conditions (NCM622: 10 mg cm^−2^; electrolyte: 20 µL; Li: 50 µm), the Li||NCM622 displays excellent electrochemical performance. Additionally, 447 Wh kg^−1^ Li||LiNi_0.8_Co_0.1_Mn_0.1_O_2_ (Li||NCM811) pouch cells have been successfully fabricated and demonstrate an exceptional cycle‐life over 150 cycles. The proposed “shielding” strategy to modulate the solvation structure paves the way for developing practical LMBs with fluorinated electrolytes.

## Graphical abstract

The solvation structure of highly fluorinated electrolyte FD (FDMA/DFEC+1.0 mol L^−1^ LiTFSI) is innovatively modulated by introducing fluorinated amide FDMA as shielding agent, whose has a higher DN value than DFEC. The FD electrolyte with this shielding effect‐regulated solvation structure facilitates uniform Li deposition, stable and conductive SEI/CEI formation, enabling the practical LMBs with high energy density and long‐cycling performance.

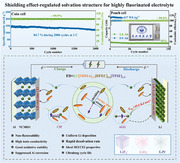

## 1 Introduction

Incumbent lithium‐ion batteries (LIBs) coupling with graphite anode can merely achieve the extreme energy densities of 200–300 Wh kg^−1^, which cannot satisfy the demands of long endurance electric vehicle developments.^[^ ^1^ ^]^ Against this background, lithium (Li) metal anode has reached the time of “revival” and will become the ultimate choice for the next generation of ≈ 400 Wh kg^−1^ high energy density battery due to its ultrahigh theoretical specific capacity (3860 mAh g^−1^) and intrinsic low electrochemical potential (−3.040 V vs standard hydrogen electrode).^[^ ^2^ ^]^ However, highly active metallic Li inevitably undergoes side reactions with the current commercial carbonated ester electrolyte to form an unstable solid electrolyte interphase (SEI), resulting in arbitrary Li dendrite electrodeposition and low Coulombic efficiency (CE) during battery cycling.^[^ ^3^ ^]^ Eventually, some knotty issues, such as the short battery cycle life and safety hazard, plague the practical applications of lithium metal batteries (LMBs).^[^ ^4^ ^]^

Electrolyte engineering strategies have been proven effective in solving the above knotty issues of LMBs.^[^ ^5^ ^]^ Historically, ethylene carbonate (EC)‐based electrolyte has opened a new era for LIBs owing to the “Magic” EC‐graphite chemistry. High DN value (16.4) of EC enables it to interact strongly with Li^+^, meaning that EC is favored in the Li^+^ primary solvation sheath.^[^ ^6^ ^]^ The electrolyte with this EC‐dominated solvation structure can form an effective SEI on graphite anode that inhibits the solvent's co‐intercalation scenario.^[^ ^7^ ^]^ Despite the booming developments of EC‐based LIBs electrolytes, the LMBs using them can only achieve a CE in the region of 70–90%, because the ion‐conducting carbonyl groups (C ═ O) in EC is chemically reactive towards Li reducing agents.^[^ ^8^ ^]^ Instead, fluorinated carbonate‐based electrolytes employing FEC or DFEC, are designed to increase the CE distinctly.^[^ ^9^ ^]^ There is a wide consensus that the selection of electronegative and weakly polarized fluorination solvents can directly inhibit the solvent coordination ability around Li^+^, which is beneficial for forming anion‐dominated solvation structures.^[^ ^10^ ^]^ Ulteriorly, the anion‐derived LiF‐rich SEI reinforces interfacial thermodynamic stability by blocking the side reactions between the Li anode and the electrolyte.^[^ ^11^ ^]^ However, LiF is indeed a salient insulator to both ionic and electronic transport, hence its presence can only generate the interphase with less conductive to Li^+^.^[^ ^12^ ^]^ Therefore, it now remains challenging to avoid the forming of over‐fluorinated interphases in fluorinated electrolyte systems equipped with anion‐dominated solvation structures. Equally important are how to tailor the “solvation‐interphase correlation” in fluorinated electrolyte, forging a thermodynamically‐dynamically bistable interphases.

Apart from interphasial kinetics, the salt solubilities and ionic conductivities of these highly fluorinated degrees of bulk electrolytes are dramatically decreased in virtue of the substantial ion aggregation and sluggish ion transport, thus hindering the operation of LMBs during their repeated cycles.^[^ ^13^ ^]^ Undoubtedly, the most straightforward tactic is to introduce cosolvent, because single solvent generally cannot furnish the perfect property parameters for achieving the competitive performances of electrolytes so far.^[^ ^14^ ^]^ Generally, high DN and low viscosity are the two most important bulk properties of the cosolvents, which determine the ion conductivities of electrolytes at the given salt concentration and temperature.^[^ ^15^ ^]^ Moreover, considering that the DN value of cosolvent can also determine the solvation sheath structure, it is possible to diminish the interaction of Li^+^‐EC, Li^+^‐FEC or Li^+^‐DFEC by hiring such cosolvent possessing the stronger coordinating ability to be engaged in the primary solvation sheath, while adding such cosolvent to further pull the FEC or DFEC away, thus driving them out of primary solvation sheath and modulating the electrochemical behavior of Li anodes.^[^ ^16^ ^]^

To target this issue, a neotype fluorinated amide, N, N‐Dimethyltrifluoroacetamide (FDMA) carrying a higher DN (22.1) and lower viscosity, is adopted to remodel the solvation sheath structure for highly fluorinated electrolyte, building a thermodynamically‐dynamically bistable interphases. Experiments and theoretical calculations demonstrate that the coordination abilities of Li^+^ with FDMA and TFSI^−^ are gradually strengthened with the augmenting of fluorination degrees of electrolytes, but the coordination abilities of Li^+^ with EC, FEC and DFEC are gradually weakened. In an optimal FD (FDMA/DFEC+1.0 mol L^−1^ LiTFSI) electrolyte, FDMA instead of DFEC, which worked as “shielding agent” preferentially partake in Li^+^ solvation sheath, avoid formation of over‐fluorinated interphases stemming from DFEC involvement. Moreover, synergistic effect of high fluorination with “shielding effect” contributes to fine interface chemistry, enabling robust SEI layer (LiF in the outer layer; Li_3_N in the inner layer; and Li_2_O in the whole layer) and thin, uniform and LiF‐contained cathode electrolyte interphase (CEI) layer. Moreover, the FDMA has no adverse impact on fluorinated electrolyte's superiority towards rapid Li^+^ desolvation behaviors, but significantly increases the high bulk ionic conductivity (3.65 mS cm^−1^). Assembled Li||Cu cell can show a high average Coulombic efficiency of 99.2% at 0.5 mA cm^−2^. Li||Li symmetric cell can be cycled stably for more than 3000 hours at 2.0 mA cm^−2^. Li||NCM622 full cell can achieve 2000 ultra‐long cycles at 1 C rate with 84.7% capacity retention and high average coulombic efficiency above 99.9%. Even under the practical conditions of high loading of NCM622 as cathode (10 mg cm^−2^), lean electrolyte (20 µL) and ultra‐thin Li foil as anode (50 µm), the Li||NCM622 full cell can retain 83.7% of its initial capacity after 200 cycles at 1 C high‐rate. More intriguingly, the assembled Li||NCM811 pouch cell can realize an ultra‐high energy density of 447 Wh kg^−1^, and a long cycle life of over 150 cycles.

## 2 Results and Discussion

### 2.1 Solvation Structure Modeling and Desolvation Analysis

**Figure 1:**
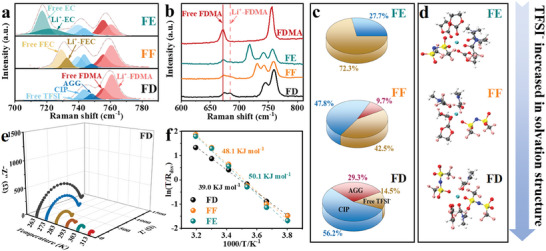
a) Raman spectra of various fluorinated electrolytes from 700 to 790 cm^−1^. b) Raman spectra of various fluorinated electrolytes and pure FDMA solvent at 600–810 cm^−1^. The solid line is the free FDMA and the dashed line is the Li^+^‐FDMA. c) Proportion of different modes of TFSI^−^ anions in various fluorinated electrolytes. d) Local snapshots are obtained from MD simulations of various fluorinated electrolytes. e) Nyquist plot of Li||Li cell when plated and stripped from 263 K to 313 K in FD electrolyte. f) Kinetic energy barriers of the Li^+^ desolvation by Arrhenius analysis.

The variations of TFSI^−^ anion solvation structures and solvent molecule coordination states in different fluorinated electrolytes are comparatively investigated using Raman spectroscopies and Fourier transform infrared spectroscopies (FTIR) (**Figure** **1**a,b; Figure S1,S2, Supporting Information). The peak area ratios of different binding states of Li^+^ to TFSI^−^ depicted in Figure 1c are derived from the fitting Raman spectra in Figure 1a. Free TFSI^−^ (≈ 740 cm^−1^) accounts for 72.3% in the FE electrolyte, and CIP (one Li^+^ coordinated to one TFSI^−^, ≈ 744 cm^−1^) accounts for only 27.7%. However, the free TFSI^−^ anions are drastically reduced to 42.5% and 14.5% in the FF and FD electrolytes, the CIPs increase to 47.8% and 56.2%, and the AGGs (two Li^+^ coordinated to one TFSI^−^, ≈ 748 cm^−1^) rise to 9.7% and 29.3%, respectively. Additionally, Figure 1d displays the snapshots of the local chemical structures derived from theoretical calculations, with one TFSI^−^ around Li^+^ in FE and FF electrolytes and two TFSI^−^ around Li^+^ in FD electrolyte. These results suggest that the increasing of TFSI^−^ anions enter the solvation sheath to form CIPs/AGGs as the increasing of fluorination degrees of electrolytes.

Moreover, the Raman peak intensities at 756 cm^−1^ and 673 cm^−1^ corresponding to the coordination abilities of Li^+^ with FDMA in various fluorination electrolytes, are also gradually strengthened with the augmenting of fluorination degrees of electrolytes. Start in the back, the coordination abilities of Li^+^ with EC, FEC and DFEC are gradually weakened, which can be further convinced by blue shifts degrees of FTIR characteristic peaks of Li^+^ solvated EC/FEC/DFEC (Figure S2a, Supporting Information). This phenomenon implies that FDMA is more easily coordinated to Li^+^ in the case of FD electrolyte, while the limited coordination space around Li^+^ results in the reducing of coordination possibilities of DFEC solvent with Li^+^.

To analyze their Li^+^ desolvation behaviors, the desolvation energies (E_dsv_) of various fluorination electrolytes are firstly estimated by fitting EIS data of Li||Li symmetric cells at various temperatures (Figure 1e,f; Figure S3, Supporting Information). The E_dsv_ for Li^+^ transfer at Li|FD interface is around 39.0 kJ mol^−1^, much smaller than that at Li|FF (48.1 kJ mol^−1^) and Li|FE (50.1 kJ mol^−1^) interfaces. Indeed, the enhancement of the participation of anion in the solvation sheath signifies the weakening of the interaction strength between the Li^+^ and dipole, thereby leading to a fast Li^+^ desolvation process at electrode/electrolyte interface.^[^ ^1^ ^]^

**Figure 2:**
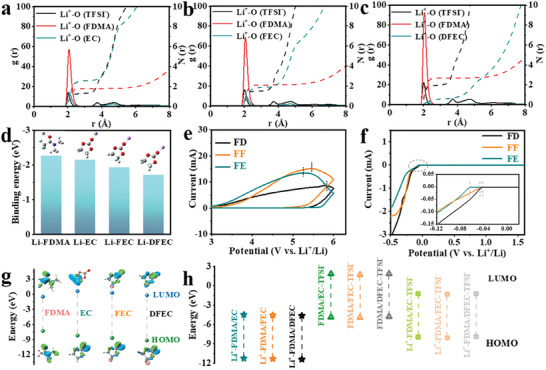
Li^+^ radial distribution functions from MD simulation of a) FE, b) FF, c) FD. d) The binding energies between Li^+^ and solvent molecules obtained by DFT calculations. e) CV curves for various fluorinated electrolytes at a scan rate of 5 mV s^−1^ on stainless steel electrodes. f) LSV curves of Li||Cu cells in various fluorinated electrolytes at a scan rate of 0.02 mV s^−1^. g) The structures and the calculated HOMO/LUMO energies of the pure solvents. h) HOMO/LUMO levels of diverse solvated complexes in various fluorinated electrolytes.

The coordination environments around Li^+^ are explored in depth by MD simulations to further analyze their Li^+^ desolvation behaviors. The model structures of various fluorinated electrolytes are established by randomly placing the number of salt and solvent molecules according to the molar ratios of the experimental formulations (Figure S4, Supporting Information). Radial distribution function (RDF) analysis assists in deciphering the distributions of solvent molecules and TFSI^−^ anions around Li^+^ in the primary solvation sheath (**Figure** **2**a–c). It is evident that the initial RDF peak of Li^+^‐F (TFSI^−^) (≈2.02 Å) is shorter than those of Li^+^‐O (all solvent molecules) in the primary solvation sheath, demonstrating that Li^+^ has a more robust binding strength to TFSI^−^ (Table S1, Supporting Information). Meanwhile, as the increasing of fluorination degrees, their coordination number of Li^+^/TFSI^−^ shows the remarkable increasing (FE: 1.242 < FF: 1.449 < FD: 1.890; Table S2, Supporting Information). Eventually, anion‐dominated solvation structure confers more CIPs and AGGs generation to FD electrolyte, which is more beneficial to the subsequent SEI formation.^[^ ^17^ ^]^

We further proceed to explore the coordination numbers of solvent molecules around Li^+^ according to the RDFs. In the FE electrolyte, the initial RDF peak of Li^+^‐O(FDMA) appears at ≈ 2.08 Å, which is shorter than Li^+^‐O(EC) in the primary solvation sheath at ≈ 2.10 Å (Figure 2a), and the total Li^+^/solvent coordination number of FE is 4.342. When switching to fluorinated electrolytes FF and FD, their initial RDF peaks of Li^+^‐O(FDMA) become shorter, appearing at around 2.06 Å, and their coordination numbers of Li^+^/FDMA increase to 2.105 for FF, 2.674 for FD (Figure 2b,c), respectively. Additionally, the initial RDF peaks of Li^+^‐O(FEC) and Li^+^‐O(DFEC) reduce to ≈ 2.12 and ≈ 2.20 Å. The total coordination numbers of FF and FD electrolytes are 3.960 and 3.237, respectively, which are significantly lower than that of FE (4.342) electrolyte (Table S2, Supporting Information). Their binding energies between Li^+^ and various solvent molecules are also in line with this investigation. As shown in Figure 2d, Li^+^ has the strongest binding energy with FDMA (−2.27 eV), and the binding energies of EC‐based solvents decrease with the increasing of the fluorination degrees (Li^+^‐EC: −2.15 eV, Li^+^‐FEC: −1.93 eV, Li^+^‐DFEC: −1.71 eV). It can be assumed prudently that FDMA due to a stronger coordinating ability with Li^+^ could preferentially enter the primary solvation sheath and occupy the space, thus shielding the FEC and DFEC molecules away from Li^+^. Based on the above, this shielding effect combined with the weak ion‐dipole interaction of the highly fluorination electrolyte contributes to a rapid Li^+^ desolvation behavior and anion‐dominated SEI formation, thus facilitating interfacial Li^+^ transfer kinetic and improving the battery performance.^[^ ^18^ ^]^

### 2.2 Thermodynamic Stability of Oxidation and Reduction

To unveil the influences of fluorination degrees of electrolytes on oxidative stabilities, the cyclic voltammetry (CV) measurements for the three electrolytes are presented in Figure 2e. FE and FF exhibit distinct oxidation peaks at 5.2 V and 5.4 V, respectively. In sharp comparison, the oxidation peak of the FD electrolyte can reach up to 5.8 V, and its peak intensity is apparently lower than that of FE and FF. This phenomenon confirms that highly fluorination effects in electrolyte systems can markedly restrict their oxidative behaviors, implying that the combination of FDMA and DFEC may competent and compatible with the high‐voltage battery system. Moreover, Figure 2f compares the linear sweep voltammetry (LSV) curves of Li||Cu cells in different fluorination electrolytes. The Li plating/stripping overpotential using the FD electrolyte (−0.044 V) is lower than that using the FF (−0.052 V) and FE (−0.068 V) electrolytes. In addition, the reduction current of the FD electrolyte is superior to that of the FF and FE electrolytes, convincingly reveals that higher fluorination degrees of solvents in electrolytes can also enable them to realize the higher reduction activities.

The electrochemical oxidative/reductive stabilities of divergent free solvents as well as solvated complexes are further analyzed by DFT calculations of the highest occupied molecular orbital (HOMO) and the lowest unoccupied molecular orbital (LUMO) (Figure 2g,h, Table S3, Supporting Information). As depicted in Figure 2g, the order of LUMO energy level is: EC (0.63 eV) > FEC (0.31 eV) > DFEC (−0.21 eV) > FDMA (−0.47 eV). Conspicuously, FDMA displays the lowest LUMO energy, and its reduced products of which have a greater opportunity to form an effective SEI. Posteriorly, DFEC with double electron withdrawing group (F) can be reduced much easier than FEC and EC, so that it could display a higher‐priority rating to participate in the formation chemistry of the SEI layer. For HOMO energy levels, the order of calculated HOMO level is: EC (−8.22 eV) > FEC (−8.70 eV) > DFEC (−9.20 eV). The DFEC possesses the lowest HOMO, which in fact extends the electrochemical stability window of FD electrolyte.

In addition to solvent molecules, various types of solvated complexes also equally affect the thermodynamic stabilities of the electrolytes (Figure 2h).^[^ ^19^ ^]^ First, the LUMO levels of Li^+^‐solvent complexes in solvation sheath are lower than those of the corresponding solvent molecules, which would then be preferentially reduced on anode surface. Secondly, their LUMO levels further increase when the TFSI^−^ (defined as CIPs: Li^+^‐solvents‐TFSI^−^) is engaged, which results in a protective SEI on anode surface. Among the various types of the solvated complexes, solvent‐TFSI^−^ complexes in three electrolytes present the highest HOMO levels, which would then be preferentially oxidative on cathode surface. Eventually, the interphases formed at electrode surface might derived from the preferentially decomposed complexes, which are further probed and illustrated in the section 2.5 and 2.6. Anyway, for various types of solvated complexes in FD electrolyte, they show a normal HOMO level close to those of FF and FE electrolytes. Therefore, FD electrolyte possesses optimal oxidation stability, in good agreement with the findings of CV and LSV tests.

### 2.3 Li Reversibility Analysis

**Figure 3:**
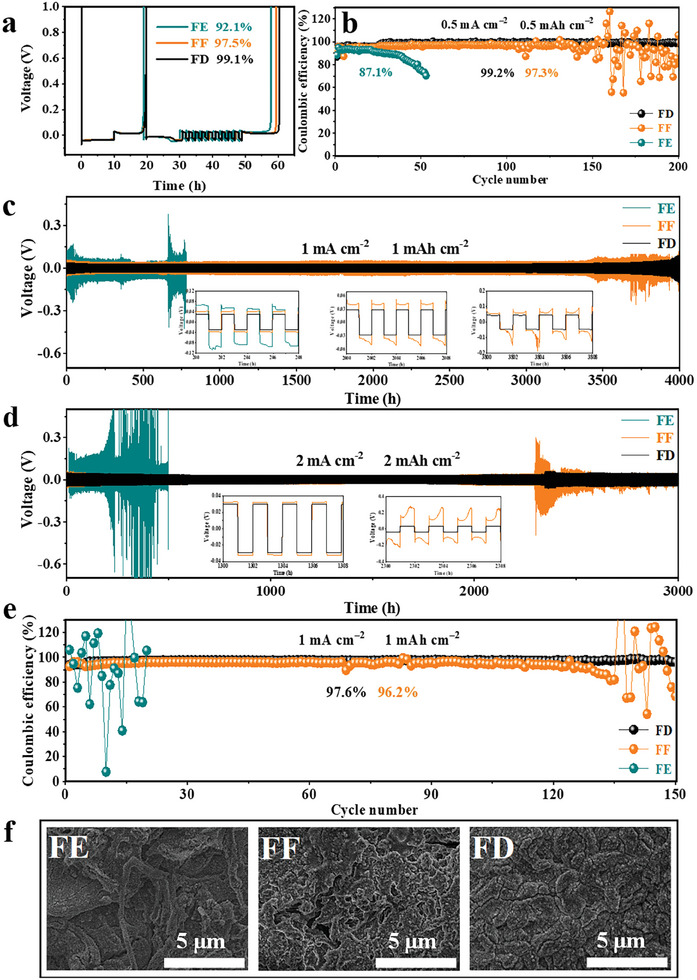
a) Li deposition/stripping CE evaluated by Aurbach measurement using Li||Cu cells. b) Li depositing/stripping CE in Li||Cu cells at 0.5 mA cm^−2^ with a capacity of 0.5 mAh cm^−2^ in various fluorinated electrolytes. c) Li||Li symmetric cells at 1.0 mA cm^−2^ under 1.0 mAh cm^−2^ in various fluorinated electrolytes. d) Li||Li symmetric cells at 2.0 mA cm^−2^ under 2.0 mAh cm^−2^ in various fluorinated electrolytes. e) Li depositing/stripping CE in Li||Cu cells at 1.0 mA cm^−2^ with a capacity of 1.0 mAh cm^−2^. f) SEM images of the Li deposition obtained by plating 5 mAh cm^−2^ Li on Cu substrates at 0.5 mA cm^−2^ in Li||Cu cells.

To explore the Li plating/stripping behavior, Li||Cu and Li||Li cells with various fluorination electrolytes are assembled upon electrochemical cycling (**Figure** **3**; Figure S5,S6, Supporting Information). Obviously, the CE and cycling stabilities of Li||Cu cells are remarkably improved with increasing fluorination. As a result, the average CE values measured by the Aurbach method suggest that FD electrolyte (99.1%) is overmatched to FF (97.5%) and FE (92.1%) in terms of Li anode's reversibility (Figure 3a). As shown in Figure 3b, the electrolyte cycling life is further verified. The Li||Cu cell based on FE electrolyte exhibits a poor average CE of 87.1% at 0.5 mA cm^−2^ under 0.5 mAh cm^−2^, with a severe CE decay after 40 cycles, due to the drastic side reaction of the EC to Li metal. Li||Cu cell with FF electrolyte has an average CE of ≈ 97.3% at 0.5 mA cm^−2^, but merely stabilizes within 150 cycles. In sharp contrast, the Li||Cu cell employing FD electrolyte can stably cycle more than 200 cycles with an average CE above 99.2%. As a comparison sample, the Li||Cu cell employing DMC/DFEC+1.0 mol L^−1^ LiTFSI only displays a relatively poor average CE of ≈95.2% during 60 cycles (Figure S5, Supporting Information) directly verifying a favorable shielding effect of FDMA. In addition to the average CE, the overpotential of Li||Cu cell with FD electrolyte (98 mV, Figure S6c, Supporting Information) is as similar as that with FF electrolyte (90 mV, Figure S6b, Supporting Information), but distinctly lower than that with FE electrolyte (161 mV, Figure S6a, Supporting Information) and DMC/DFEC+1.0 mol L^−1^ LiTFSI (174 mV, Figure S6d, Supporting Information). This trend is also in line with the results of Li||Li symmetric cells. In Figure 3c and Figure 3d, the FD electrolyte can be stabilized for more than 4000 h and 3000 h at current densities of 1.0 mA cm^−2^ and 2.0 mA cm^−2^, respectively, with overpotentials of only ≈29 mV and ≈30 mV, which outperforms the FE electrolyte (700 h at 1.0 mA cm^−2^ with the considerable large overpotential of 67 mV;) and even FF electrolyte (3400 h at 1.0 mA cm^−2^ with overpotential of 43 mV; 2300 h at 2.0 mA cm^−2^ with overpotential of 33 mV). This phenomenon may be interpreted as the synergistic advantage of FD electrolyte with the high bulk ionic conductivity and thermodynamically stable SEI layer, as discussed later. Most significantly, even at a higher current density of 1.0 mA cm^−2^ under 1.0 mAh cm^−2^ (Figure 3e), the Li||Cu cell employing the FD electrolyte can still show a superior reversibility and the highest CE of 97.6% during 150 cycles, which is much higher than most previous reports.^[^ ^20^ ^]^

Furthermore, their Li deposited morphologies are compared by scanning electron microscopy (SEM) images, which obtained by plating 5 mAh cm^−2^ Li on Cu substrate at 0.5 mA cm^−2^ (Figure 3f). In FE electrolyte, the deposited Li displays the numerous elongated dendrites and irregular pits, resulting in a serious security risk and inferior Li reversibility. In FF electrolyte, the deposited Li remains loose and porous structure without obvious dendrite morphology, whose unavoidably gives rise to the continued SEI regeneration derived from the film‐forming functional FEC. In sharp contrast, the Li deposited in the FD electrolyte exhibits a homogeneous and compact surface morphology. The thickness of the deposited Li is also observed by cross‐sectional SEM. The Li deposition thicknesses of FE and FF are 65.7 µm and 45.4 µm, respectively (Figure S7a,b, Supporting Information). The porous Li layer with high surface area is in direct contact with the electrolyte, resulting in decomposition of electrolyte and the loss of active Li associated with decreased CE. Thicker deposited Li layer may adversely affect Li^+^ migration, causing increased internal resistance and deteriorating the kinetic characteristics of the cells.^[^ ^21^ ^]^ Encouragingly, the Li deposition in the FD electrolyte has good densification, with a Li layer thickness of only 38.9 µm, enabling high CE and long cycle stability of the battery (Figure S7c, Supporting Information). These Li||Cu and Li||Li cells results are also consistent with the aforementioned solvation structure analyses, indicating that the solvation structure of the highly fluorinated electrolyte can be tactfully tailored by introducing shielding agent FDMA, thereby realizing a fast Li^+^ desolvation rate and thermodynamically stable interphases, simultaneously.

### 2.4 Full Cells Electrochemical Performance

**Figure 4:**
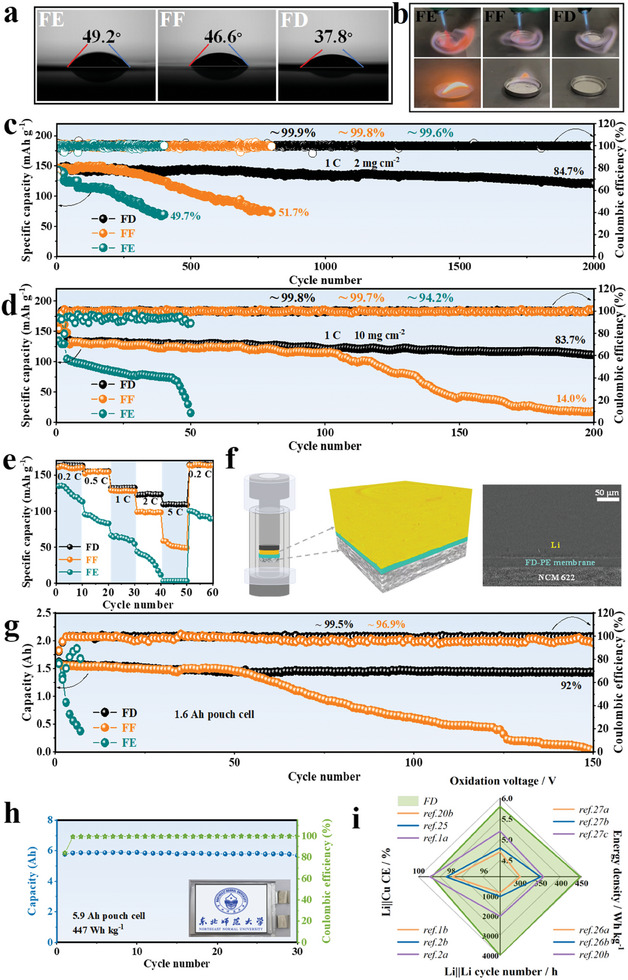
a) Contact angle tests of various fluorinated electrolytes. b) Flammability tests of various fluorinated electrolytes. c) Cycling and CE comparisons of Li||NCM622 (loading mass: 2 mg cm^−2^) coin cells in various fluorinated electrolytes. d) Cycling and CE comparisons of Li||NCM622 (loading mass: 10 mg cm^−2^) coin cells in various fluorinated electrolytes. e) Rate capabilities of the Li||NCM622 coin cells between 2.8–4.3 V at different C‐rates. f) Cell schematic from X‐ray tomography of the FD electrolyte, 3D rendering of the cell interior (yellow part: Li anode; green part: PE separator; gray part: NCM622 cathode) and reconstructed X‐ray tomography slice. g) Cycling performances and CEs of Li||NCM811 pouch cells at 1.6 Ah in various fluorinated electrolytes. h) Cycling performance and CE of Li||NCM811 pouch cell at 5.9 Ah in FD electrolyte. The inset is the optical image of the corresponding pouch cell. i) Radar characterization plots of FD electrolyte and the representative electrolyte systems reported in previous literatures.

To evaluate the commercializing possibility, key physical properties of these fluorination electrolytes that the use of LiTFSI salts as solute, such as ionic conductivity, wettability, flammability and anodic behavior of Al current collector are primarily tested, and the results are depicted in **Figures** **4**a,b and S8–S10 (Supporting Information). The ionic conductivities of EC, FEC and DFEC with 1 mol L^−1^ LiTFSI are 0.80, 0.39 and 0.26 mS cm^−1^, respectively, which conform to the order of their DN values (Table S4, Supporting Information). After introducing FDMA as cosolvent, all the prepared fluorination electrolytes show an order of magnitude higher ionic conductivities than their corresponding sole solvent electrolytes, which is attributed to the FDMA's high DN value (22.1) induced high lithium salt dissociation. Among them, FD electrolyte presents the lowest contact angles (37.8°) presumably due to the relatively low viscosity of DFEC solvent, which can optimize the wettability of the PE membrane and further assist in minimizing the cell's interfacial impedance.^[^ ^22^ ^]^ Moreover, the self‐extinguishing time (SET) of the designed FD electrolyte (0 s g^−1^) is remarkably shorter than that of the FE electrolyte (46 s g^−1^) and the FF electrolyte (25 s g^−1^), manifesting that the higher degree of fluorine substitution in the solvents can render the electrolyte absolutely non‐flammable.^[^ ^23^ ^]^ To investigate the Al corrosion, the CV measurements of Li||Al cells in various electrolytes are interrogated and shown in Figure S9 (Supporting Information). The CV curve of FE electrolyte presents a typical counter‐clockwise current loop characteristic, the anodic current begins to increase abruptly above 4.0 V during the first anodic scan, and then increases dramatically during following cycles, implying a continuous corrosion of the Al current collector (Figure S9a, Supporting Information). In contrast, both FF and FD electrolytes show the clockwise current loop characteristic (Figure S9b,c, Supporting Information). The major differences are found in their anodic current response. Compared to FF, FD electrolyte displays a weaker and more stable anodic current response during the following cycles. Subsequently, the Li||Al cells are disassembled after CV cycling and the corresponding SEM images of Al foil are collected (Figure S10, Supporting Information). As shown in Figure S10b (Supporting Information), the surface of the Al collector in the FE electrolyte is severely corroded. This phenomenon is significantly ameliorated with increasing fluorination of the electrolyte. There is slight corrosion occurred on the Al surface in the FF electrolyte (Figure S10c, Supporting Information), but almost negligible change on the Al foil in the FD electrolyte (Figure S10d, Supporting Information). The above results confirm that the Al collector is considerably stable in application of FD electrolyte.

Aiming to evaluate the electrochemical performances of the various fluorination electrolytes, Li||NCM622 (loading mass: 2 mg cm^−2^) coin cells are first assembled and measured at 1 C rate (Figure 4c). The Li|FE|NCM622 is concomitant with continuous capacity decay to 49.7% within 400 cycles. Li|FF|NCM622 exhibits a capacity decline at 414^th^ cycle and only retains 51.7% of the initial capacity at 800^th^ cycle. By contrast, Li|FD|NCM622 demonstrates an outstanding ultralong cycle‐life of 2000 cycles with an impressive capacity retention of 84.7% and a high average CE above 99.9%. Furthermore, long‐term cyclabilities tested at extreme conditions are considered as one of the challenging aspects for practical LMBs. To prove the superiority of FD electrolyte, we conduct the electrochemical performance tests of full cells assembled with high‐loading NCM622 cathode (10 mg cm^−2^), ultrathin Li anode (50 µm) and lean electrolyte (20 µL) at 1 C rate in the voltage range of 2.8‐4.3 V (Figure 4d; Figure S11,S12, Supporting Information). The Li|FE|NCM622 demonstrates a poor capacity retention (72.5%) even at the initial 40 cycles, and a rapid capacity degradation during the subsequent cycles. The Li|FF|NCM622 electrolyte only sustains a capacity retention of 14% after 200 cycles, and the capacity diving occurs at 117^th^ cycle. As stark contrast, the Li|FD|NCM622 displays the best capacity retention of 83.7% at 1 C even after 200 cycles. This result is also superior to the compared sample DMC/DFEC+1.0 mol L^−1^ LiTFSI (the initial specific capacity is only 83 mAh g^−1^; capacity retention of 45.8% after 100 cycles), owing to the formation of unique shielding effect‐regulated solvation structure for FD electrolyte (Figure S11, Supporting Information). As shown in the voltage profiles within 200 cycles (Figure S12, Supporting Information), the Li|FD|NCM622 possesses the minimum overpotential oscillation, in accordance with its impedance evolution (Figure S13, Supporting Information). This result is one of the most impressive cycling performances in liquid electrolyte‐based LMBs in comparable conditions (Table S5, Supporting Information).

The High‐rate performance of Li||NCM622 cells are also evaluated. When a current rate of 2 C, the FE electrolyte shows a serious capacity degradation within 10 cycles. The FF electrolyte only exhibits ≈100 mAh g^−1^ capacity, while the FD electrolyte still maintains a high capacity of ≈124 mAh g^−1^. This trend continues at a further high current rate of 5 C. While FF electrolyte gradually degrades to 49 mAh g^−1^ within 10 cycles, the FD electrolyte shows superior rate capability and delivers 110 mAh g^−1^ at 5 C (Figure 4e). Such outstanding high C‐rates cyclability of the proposed FD electrolyte is closely associated with its unique solvation structure, which endows FD with a fast desolvation rate and optimal kinetic stability.

To measurably probe the internal morphology of the Li||NCM622 cell cycled with FD electrolyte, the synchrotron radiation X‐ray tomography is adopted, as delineated in Figure 4f. It can be observed that the cycled Li‐metal surface still exhibits a dense and homogeneous morphology, and the NCM622 side displays an intact and crack‐free uniform particle distributions, thereby ensuring a long‐term cycle stability of FD electrolyte.^[^ ^24^ ^]^

Additionally, the Ah‐level Li||NCM811 pouch cells are manufactured and tested under more stringent conditions to further validate the commercialization potential of FD electrolyte (Figure 4g,h; Figure S14, Supporting Information). The specific assembly conditions of the pouch cells are listed in Table S6 (Supporting Information), the designed a Li||NCM811 pouch cell only has a N/P = 2.38, in which the electrolyte amount is as low as 2 g Ah^−1^ and the cathode loading of NCM811 is as high as 20 mg cm^−2^. Obviously, the FE electrolyte cannot sustain the 1.6 Ah pouch cell operation. The FF electrolyte pouch cell undergoes a rapid capacity fade over 50 cycles, with an average CE of about 96.9%. Encouragingly, the FD pouch cell possesses a prominent cycling stability, maintaining 92% of its initial capacity after 150 cycles with a high average CE of 99.5%, and displaying the lowest polarization voltage (Figure S14, Supporting Information). Even if the designed capacity of pouch cell reaches 5.9 Ah, the FD‐based pouch cell can perform 30 cycles without capacity degradation, and achieve an ultrahigh energy density of 447 Wh kg^−1^ (Figure 4h). Compared with the oxidation voltage,^[^ ^25^ ^]^ compatibilities with Li anode,^[^ ^26^ ^]^ CE of Li||Cu cell, and energy density of pouch cell for representative reported electrolytes,^[^ ^27^ ^]^ the FD electrolyte presents multiple advantages, which is promising to be a safety and high‐efficient electrolyte for realizing high energy density LMBs applications (Figure 4i).

### 2.5 SEI Layers and CEI layers

**Figure 5:**
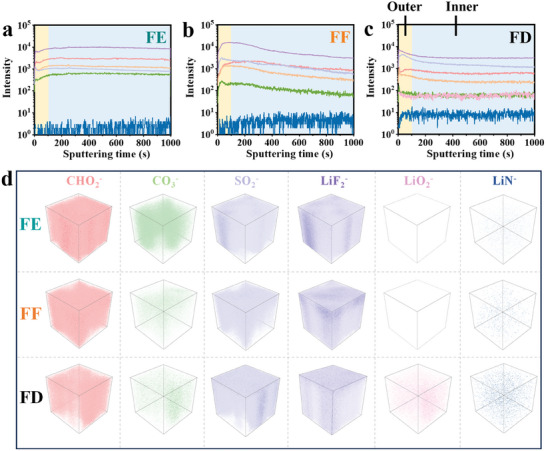
Depth profiles of selected various secondary ion fragments on the cycled Li anodes surface when cycling in a) FE, b) FF, and c) FD electrolytes from the time‐of‐flight secondary ion mass spectrometry (TOF‐SIMS) measurements, with the curve colors corresponding to the ion fragments. d) Corresponding 3D visual images of various fragments when using FE, FF and FD electrolytes.

To unlock the “solvation‐interphase correlation” in the prepared fluorinated electrolytes, the SEI and CEI properties of the cycled high‐voltage NCM622 cathode and Li anode surface are evaluated, respectively. The variations of the SEI layers and the distributions of by‐products at the cycled Li surfaces are meticulously characterized using high‐sensitivity time‐of‐flight secondary ion mass spectrometry (TOF‐SIMS) combined with X‐ray photoelectron spectroscopy (XPS) (**Figure** **5**; Figures S15–S18, Supporting Information). Representative secondary ion fragments of organic species (CHO_2_^−^ and C_2_HO^−^) and inorganic species (CO_3_^−^, SO_2_^−^, LiF_2_^−^, LiO_2_^−^ and LiN^−^) are selected to analyze the interphasial products of the various fluorination electrolytes. Firstly, the 3D TOF‐SIMS mapping images of top‐down depth sputtering indicate that the FE electrolyte contains large‐scale CHO_2_^−^, C_2_HO^−^ and CO_3_^−^, which are mainly imputed to the continuous EC decomposition (Figure 5d; Figure S15, Supporting Information). Satisfyingly, this drastic solvent decomposition situation can be effectively suppressed by enhancing the fluorination degrees of the carbonate‐based solvents. Apparently, the contents and distributions of these species in the FF and FD electrolytes are much lower than those in the FE electrolyte (Figure 5b,c). Second, SO_2_^−^ fragments are derived from the reduction decomposition of TFSI^−^ anions, and the FD electrolyte exhibits the highest ratio of SO_2_^−^ species, suggesting a stronger tendency of decomposition of the TFSI^−^ anions, which is correlated to more TFSI^−^ participation into its primary solvation sheath of Li^+^. This is consistent with the C1s spectrum of XPS, in which strong C‐SO_x_ signal can be observed in the SEI layer formed in FD electrolyte (Figure S16, Supporting Information). Thirdly, 3D TOF‐SIMS mapping images of all three electrolytes show LiF_2_^−^ fragments throughout the SEI layers, proving the LiF‐rich SEI typically generated in the fluorination electrolytes. However, the LiF_2_^−^ distributions in the FF and FE electrolytes are uneven, resulting in a fast Li and electrolyte depletion. Furthermore, FE electrolyte gains the more LiF_2_^−^ fragments compared to FF and FD electrolytes in whole sputtering range, with higher LiF contents (Figure S18, Supporting Information). Aside from TFSI^−^, LiF_2_^−^ fragments originated from the massive and continuous decomposition of fluorination solvents should be taken into consideration. Therefore, the over‐accumulated LiF in FE electrolyte can be partially derived from the violent decomposing of FDMA, which can be further testified by the detecting LiF in XPS F1s spectrum collected from the cycled Li surface in FDMA/LiClO_4_ electrolyte (Figure S19, Supporting Information). But in FD electrolyte, only relatively high content and uniform distribution of LiF_2_^−^ fragments are detected in the outer SEI layer (the sputtering time of 100 s), as verified in Figure 5c, which can availably prevent the Li dendrite piercing due to the ultrahigh mechanical strength of LiF (55.1 GPa).^[^ ^28^ ^]^ In its inner layer of SEI, LiN^−^ fragment corresponding to Li^+^ conducting Li_3_N, is uniformly distributed.^[^ ^29^ ^]^ Nevertheless, the Li_3_N contents both in FE and FF electrolytes are relatively low, whose visibly increases as the fluorination degree of solvents increases. Last but most important, the LiO_2_^−^ fragment corresponding to a good Li^+^ conducting/e^−^ non‐conducting Li_2_O can also be found throughout the SEI layer formed in FD electrolyte, which may be stemmed from the decomposition of “free” DFEC solvent.^[^ ^30^ ^]^ This finding likewise confirms the joint advantages of shielding agent effect of FDMA and weakly solvation power of DFEC in FD electrolyte. Overall, the promoting of the massive TFSI^−^ decomposition, the limited FDMA degradation, and the appearance of free‐state DFEC decomposition products result in a dense and smooth SEI layer (LiF in the outer layer; Li_3_N in the inner layer; Li_2_O throughout the whole layer) formed on the Li anode in optimal FD electrolyte.

To gain insight into the effect of SEI formation on the microstructure of the electrodes, the cycled Li anodes (disassembled on 30‐cycle Li||NCM622 coin cells) are collected for SEM analysis (Figure S20, Supporting Information). On the surface of the Li anode of the FE electrolyte, the concave and convex surfaces are generated by dead Li deposition and massive Li dendrites, causing a disconnection of the conduction path of Li^+^ on Li anode, and rapidly failing the battery. This drawback is obviously ameliorated after solvent fluorination, and only a little portion of dead Li distribution can be detected in the FF electrolyte, while the Li anode surface of the FD electrolyte remains intact after cycling. The above test results confirm that the SEI layer supplied by the FD electrolyte efficaciously suppresses the interaction between the Li and the electrolyte as well as the growth of Li dendrites, minimizes the loss of the Li stock, and improves the cycling performance of the Li electrode.

The CEI layer properties on NCM622 cathode surface in various fluorination electrolytes are characterized by transmission electron microscopy (TEM) and XPS (Figures S21–S25, Supporting Information). The thicknesses of CEI layer are in the following order: 22 nm (FE) > 11 nm (FF) > 5 nm (FD). Both FE and FF electrolyte display the heterogeneous thicknesses of CEI layers (Figure S21a,b, Supporting Information). In comparison, the FD electrolyte provides a suitable thinner and homogeneous CEI layer in the coverage of NCM622 cathode surface (Figure S21c, Supporting Information), which can not only decrease cell polarization during cycling, but also restrict severe corruption of the NCM622 cathode by isolating the electrolyte. As shown in Figure S23 (Supporting Information), the Li1s spectra of XPS results indicate that the CEI components of all three fluorination electrolytes are mainly composed of LiF, which has a large oxidative stability and excellent mechanical stability, accounting for their wide electrochemical stable window (> 5.0 V).^[^ ^31^ ^]^ Discriminatingly, FE electrolyte only endows NCM622 cathode with the minimal LiF accompanied by the abundant organic components (CF_3_, Poly (CO_3_), ROCO_2_Li), which is unbeneficial for the protection of the cathode (Figure S25a, Supporting Information).^[^ ^32^ ^]^ With the improvement of the fluorination degrees of solvents, LiF content gradually augments and the organic component content decreases in CEI layers of FF and FD electrolytes, suggesting that the electrolyte oxidation decomposition can be effectively controlled by introducing more F‐substituted solvents. These results demonstrate that the synergistic interaction between FDMA and DFEC affords a homogeneous and stable CEI protective layer, which can boost the high voltage cycling longevities of the full cells.

### 2.6 Solvation‐Interphase Correlation

**Figure 6:**
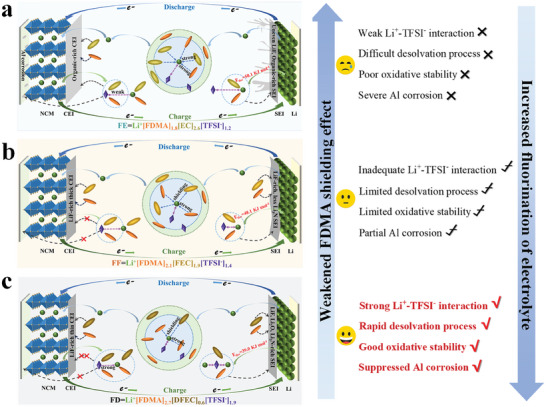
Schematic diagrams of the primary solvation sheaths in a) FE, b) FF and c) FD electrolytes and the solvation evolutions at the cathodes and anodes of the cells.

To establish the schematic diagrams of the dynamic evolutions of the solvation structures of the various fluorination electrolytes during cell's charging and discharging, the equation Li^+^[solvent1]_x_[solvent2]_y_[TFSI^−^]_z_ is adopted to represent primary solvation sheath and explain the solvation‐interphase correlation (**Figure** **6**a–c). The aforementioned convincing evidences came from the Raman experimental results and DFT theoretical calculations directly indicate that fluorinated solvent in the electrolyte can enable more TFSI^−^ anions to enter the primary solvation sheath, forming more CIPs/AGGs and thus impairing the interactions between Li^+^ and solvent molecules. In the FE electrolyte, the interaction of Li^+^ with TFSI^−^ is weaker than that with EC solvent molecule, thus “free” TFSI^−^ anion concentration increases, accelerating the corrosion of Al foil.^[^ ^33^ ^]^ Simultaneously, a myriad of FDMA/EC‐TFSI^−^ complexes with the lower HOMO energies are generated, resulting in an unstable and incomplete CEI layer with aggregated organic components on the cathode surface (Figure 6a).

With the increasing of fluorination degrees of electrolytes, the interaction between Li^+^ and TFSI^−^ is enhanced, effectively inhibiting the corrosion of Al foil and the decomposition of electrolyte. Notably, the neutralization of Li^+^ with TFSI^−^ in the FF electrolyte is insufficient, and FDMA/FEC‐TFSI^−^ decomposition inevitably exists, leading to an excessively thick CEI layer formation and a slow detachment of Li^+^ from the cathode (Figure 6b). However, the DFEC in the FD electrolyte possesses a weaker binding ability to Li^+^, and the TFSI^−^ anions basically coordinate to Li^+^, thus the abundant “free” DFEC exists on the cathode surface. In the meantime, the higher DN value cosolvent FDMA could quickly coordinate with Li^+^ and then migrates to the anode side once a new Li^+^ releases from the cathode side, implying that relatively few FDMA is involved in the formation of CEI layer. Therefore, FD electrolyte endows high‐voltage cathode surface with a thin and LiF‐moderate CEI layer (Figure 6c).

On the anode side, the Li^+^ is more surrounded by EC and FDMA solvent molecules of the FE electrolyte (FE = Li^+^[FDMA]_1.8_[EC]_2.6_[TFSI^−^]_1.2_). The consequent high desolvation energy barrier (E_dsv_ = 50.1 kJ mol^−1^) makes it extremely hard to deposit Li onto the Li anode, which leads to Li dendrite growth (Figure 1f).^[^ ^34^ ^]^ The formed Li^+^‐FDMA/EC complexes are violently reductive, and the produced SEI layer containing abundant organic components and uneven LiF, failing to restrain the persistent decomposition of the EC. Conspicuously, the interaction between Li^+^ and TFSI^−^ of FD electrolyte is progressively strengthened in virtue of weak solvation ability of fluorination solvent, and the more CIPs and AGGs are preferentially reduced to form SEI layer containing LiF and Li_3_N. Synchronously, the interaction between Li^+^ and DFEC is extremely weak, and “free” DFEC molecules participate in the formation of the Li_2_O interphasial ingredients. Benefiting from the FD's fast desolvation rate, Li^+^ can be rapidly detached from the solvation structure and deposited uniformly on the Li anode surface. By comparison, FF electrolyte with the relatively strong Li‐solvent interaction and moderate desolvation rate results in an inhomogeneous Li deposition and over‐fluorinated interphases on the Li anode surface. Overall, the solvation structure of highly fluorination FD electrolyte can be exactly tailored by introducing FDMA as shielding agent, promising an ethereal interfacial property for practical application of LMBs.

## 3 Conclusion

A bran‐new highly fluorination electrolyte (FDMA/DFEC with 1.0 mol L^−1^ LiTFSI) is developed through manipulating solvation structure by introducing high DN value FDMA as shielding agent to realize both high bulk ionic conductivity and rapid interphasial kinetics. FDMA molecules carrying the stronger coordinate abilities of Li^+^ pioneeringly occupy sites in primary solvation sheath, thus driving DFEC away and modulating SEI/CEI components. Ultimately, both assembled asymmetric and symmetric cells gain stable long‐term cyclability with superior rate performance, outperforming both EC‐ and FEC‐based electrolytes. Most strikingly, the Li||NCM811 pouch cell displays an ultrahigh energy density of 447 Wh kg^−1^ and stable operation over 150 cycles, showing a promising potential for development and application of LMBs. This study brings a new avenue for the development of fluorination electrolytes and the proposed shielding strategy would also be applicable to other electrolyte systems in energy storage devices.

## 4 Experimental Section

### Electrolytes Preparation

Ethylene carbonate (EC, 99%), fluoroethylene carbonate (FEC, 99%) and dimethyl carbonate (DMC, 99%) were purchased from Aladdin. N,N‐Dimethyltrifluoroacetamide (FDMA,>98%) and bistrifluoromethanesulfonimide lithium salt (LiTFSI) were purchased from Sigma‐Aldrich. The trans‐4,5‐Difluoroethylenecarbonate (DFEC, 98%) was bought from Zhengzhou Alfa Chemical Co., Ltd. All solvents were dried on molecular sieves for 48 h, and the electrolytes were prepared in an Ar‐filled glove box, in which the contents of O_2_ and H_2_O were strictly maintained below 0.1 ppm. The electrolyte was prepared by dissolving 1 mol L^−1^ LiTFSI in the solvent mixtures of FDMA and EC (1:1 by volume, denoted as FE). Replace EC with FEC and DFEC, named FF and FD, respectively. The solvent volume ratio in the DMC/DFEC+1.0 mol L^−1^ LiTFSI electrolyte was 1:1. Li foils with the thickness of 50 µm and 400 µm were ordered from China Energy Lithium Co. Ltd and stored in an Ar‐filled glove box.

The Li [Ni_0.6_Co_0.2_Mn_0.2_] O_2_ (NCM622) cathode was prepared by muddy mixture containing 80 wt. % NCM622 powder, 10 wt. % Super P and 10 wt. % PVDF (polyvinylidene difluoride) in a moderate amount of NMP (N‐Methylpyrrolidone). The obtained slurry was coated on Al foil, followed by drying 12 h in a vacuum oven at 120 °C. The average mass loading of the NCM622 cathode was calculated as 2 ± 0.1 mg cm^−2^ and 10 ± 0.1 mg cm^−2^.

### Electrochemical Measurements

Using CR2025 coin‐type assembly Li||Li symmetrical cells, Li||Cu asymmetrical cells and Li||NCM622 full cells. The coin cells assembly was performed in an Ar‐filled glove box. The cathode and anode were separated by PE separator soaked with quantitative electrolyte (Li||NCM 622 (2 mg cm^−2^): 30 µL, Li||NCM622 (10 mg cm^−2^): 20 µL, Li||Li: 35 µL, Li||Cu: 35 µL). 50 µm Li foil was used for the Li||NCM622 (10 mg cm^−2^) full cells and Li||Cu cells, and 400 µm Li foil was used for the Li||NCM 622 (2 mg cm^−2^) full cells and Li||Li cells. The Li||Cu coin cells were galvanostatically cycled at current density of 0.5 and 1.0 mA cm^−2^, capacity was 0.5 and 1.0 mAh cm^−2^, and the cut‐off voltage was 1.0 V. The Li||Li coin cells were cycled at 1.0 and 2.0 mA cm^−2^ with capacity of 1.0 and 2.0 mAh cm^−2^. Li||NCM622 fresh cell was first activated in 3 cycles, and then cycled at 1 C rate with the voltage range was 2.8–4.3 V. All the coin cells and pouch cells were tested using Neware battery test system (CT‐4008Tn‐5V10mA‐164, Shenzhen, China) at room temperature. In the linear scanning voltammetry (LSV) test of Li||Cu cell, the voltage range was 0–1.6 V with a scan rate of 0.02 mV s^−1^. The cyclic voltammetry (CV) test of stainless‐steel electrode was performed with a voltage range of 2.8–6.0 V at a scan rate of 5.0 mV s^−1^. The CV test of Li||Al cell was performed with a voltage range of 3.0–4.3 V at a scan rate of 5 mV s^−1^. Electrochemical impedance spectroscopy (EIS) was performed with a frequency range of 100 kHz to 10 mHz with a sinusoidal amplitude of 10 mV. All curves were performed on a PARSTAT 4000 electrochemical workstation.

EIS tests of Li||Li cells were performed at −10 – 40 °C (263–313 K), and the energy barrier for desolvation (*E_dsv_*) was calculated by fitting an equivalent circuit model through the Arrhenius equation:

(1) $$k=\frac{T}{R_{dsv}}=A\exp\left(-\frac{E_{dsv}}{RT}\right)$$

where *k* was the rate constant, *T* was the absolute temperature, *R_dsv_* was the charge transfer resistance, *A* was the pre‐exponential constant, *E_dsv_* was the activation energy of charge transfer, and *R* was the standard gas constant.

Li||NCM811 pouch cells were assembled in a dry room (dew‐point temperature: −40 °C) at room temperature. The commercial NCM811 cathode was mixed with polyvinylidene fluoride (PVDF) binder and super‐P carbon with a mass ratio of 97.5:1.5:1 in N‐Methyl‐2‐pyrrolidone (NMP) solvent. The obtained slurry was scraped onto an Al foil collectors, followed by drying at 120 °C for 12 h in vacuum. Eventually, the active material loading of NCM811 cathode was about 20 mg cm^−2^. The areal capacity of NCM811 cathode was 3.8 mAh cm^−2^. The thickness of Li foil was 50 µm. First, the cathode and anode were stacked layer by layer using PE separator. Second, Ni and Al pole lugs were welded to Cu and Al collectors, respectively, by ultrasonic spot welding. Third, the cell was packed with aluminum‐plastic film. Finally, a quantitative electrolyte was injected into the pouch cell, and assembled pouch cell was aged for 24 h at 60 °C and followed by degassing step. After degassing process, formation cycles were performed with a 0.2 C rate in a voltage range of 2.5–4.2 V.

### Materials Characterization

The ionic conductivity of the electrolytes was measured using a conductivity meter (DDS‐307A). The contact angles (SURFTENS) of various electrolytes were detected by the penetration of the electrolytes into the PE membrane. The self‐extinguishing time (SET) of various electrolytes were tested to investigate the flammability of the electrolyte. The Li anode surfaces morphology and cathode active particle morphology were observed using scanning electron microscopy (SEM) and transmission electron microscopy (TEM). The solvation structures of the electrolytes were analyzed using Fourier transform infrared spectroscopy (FTIR) (Nicolet 6700, Thermo Fisher Scientific) and Raman spectroscopy (Horiba JobinYvon T64000). Synchrotron X‐ray tomography results were obtained by processing on avizo studio 2021 software. X‐ray photoelectron spectroscopy (XPS) (ESCA Lab 220XL) was used to identify the phase relationship between the electrolytes and the electrodes. Time‐of‐flight secondary ion mass spectrometry (TOF‐SIMS) (ION‐TOF GmbH, Germany) was used to characterize the composition and distribution of SEI on the Li anodes. The cycled Li anodes were sputtered with a 1 keV Cs^+^ ion beam with a sputtering area of 290 µm × 290 µm.

### Computational Details

DN calculations were performed using Gaussian with the B3LYP method and the def2‐SVP basis set. The ground state geometries were optimized by density functional theory (DFT) based on single crystal structures. The van der Waals (vdW) interactions were described with the DFT‐D3 method in Grimme's scheme. The structures were visualized with VESTA.

The partial charge of LiTFSI, FDMAC, EC, FEC and DFEC molecule was calculated using Gaussian 16 code and the 6–311 g (d, p) basis functions were applied. The OPLSS‐AA force field and MKTOP were used to parametrize all atoms, such as the bond parameters, angle parameters and the dihedral angles, and so on. The coordination structures of solvents, Li^+^ and TFSI^−^ in different electrolytes were simulated by molecular dynamics (MD) simulation. In system FE, the monomer ratio of LiTFSI: FDMAC: EC = 1 mol: 4.33 mol: 7.51 mol and 50 LiTFSI, 217 FDMAC and 375 EC molecule were randomly inserted into a cube box with a side length of 6.0 nm; In system FF, LiTFSI: FDMAC: FEC = 1 mol: 4.33 mol: 6.86 mol and 50 LiTFSI, 217 FDMAC and 343 FEC molecule were randomly inserted into a cube box with a side length of 6.0 nm; In system FD, LiTFSI: FDMAC: DFEC = 1 mol: 4.33 mol: 6.13 mol and 50 LiTFSI, 217 FDMAC and 306 DFEC molecule were randomly inserted into a cube box with a side length of 6.0 nm, too. The MD simulations were performed in the GROMACS 2021 software package. The steepest descent method was applied to minimize the initial energy for each system with a force tolerance of 1.0 kJ (mol^−1^ nm^−1^) and a maximum step size of 0.002 ps before MD calculations. In all the three directions, periodic boundary conditions were imposed. Leapfrog algorithm was used to integrate the Newtonian equation of motion. The MD simulation was processed in an NPT ensemble and the simulation time was 20 ns. In NPT simulations, the pressure was maintained at 1 bar by the Berendsen barostat in an isotropic manner and the temperature was maintained by the V‐rescale thermostat at 298.15 K. The Particle‐Mesh‐Ewald (PME) with a fourth‐order interpolation was used to evaluate the electrostatic interactions and the grid spacing was 1.0 Å, whereas a cutoff of 1.0 nm was employed to calculate the short‐range van der Waals interactions.

The density functional calculations presented herein were completed with the B3LYP functional in combination with the D3BJ dispersion correction as implemented in the Gaussian 16 software. The 6–311G (d, p) basis set was adopted for the geometry optimization and frequency calculations. The geometries were fully optimized without any structural constraints. The binding energy (E_b_) was calculated by the following equation:

(2) $$E_{b}=E_{Complex}\;-\left(E_{M_{1}}+E_{M_{2}}\right)$$

where *E_Complex_*, *E_M1_*, and *E_M2_* represent the energies of the complex, and energies of the interacting molecules, respectively. The molecular orbital figures were extracted from the Multiwfn 3.8 program and visualized using the VMD software.

## Conflict of Interest

The authors declare no conflict of interest.

## Supporting information



Supporting Information

## Data Availability

The data that support the findings of this study are available from the corresponding author upon reasonable request.
