# Supplementary material for: Remodeling Highly Fluorinated Electrolyte via Shielding Agent Regulation toward Practical Lithium Metal Batteries
Source: Adv Sci (Weinh). 2024 Oct 10;11(45):2404248. doi: 10.1002/advs.202404248 (PMC11615804; doi:10.1002/advs.202404248)
Supplement: Supplementary file 1 — Supporting Information [file ADVS-11-2404248-s001.docx]

Supporting Information

**Remodeling Highly Fluorinated Electrolyte via Shielding Agent Regulation towards Practical Lithium Metal Batteries**

*Yutong Yang, Shunchao Ma, Hongxing Yin, Yanan Li, Silin Chen, Yu Zhang, Dan Li, Feilong Dong, Yue Zhang, Haiming Xie, * and Lina Cong**

**Figure S1**. Raman spectra of various fluorinated electrolytes and pure FDMA solvent at 500-1300 cm^-1^.


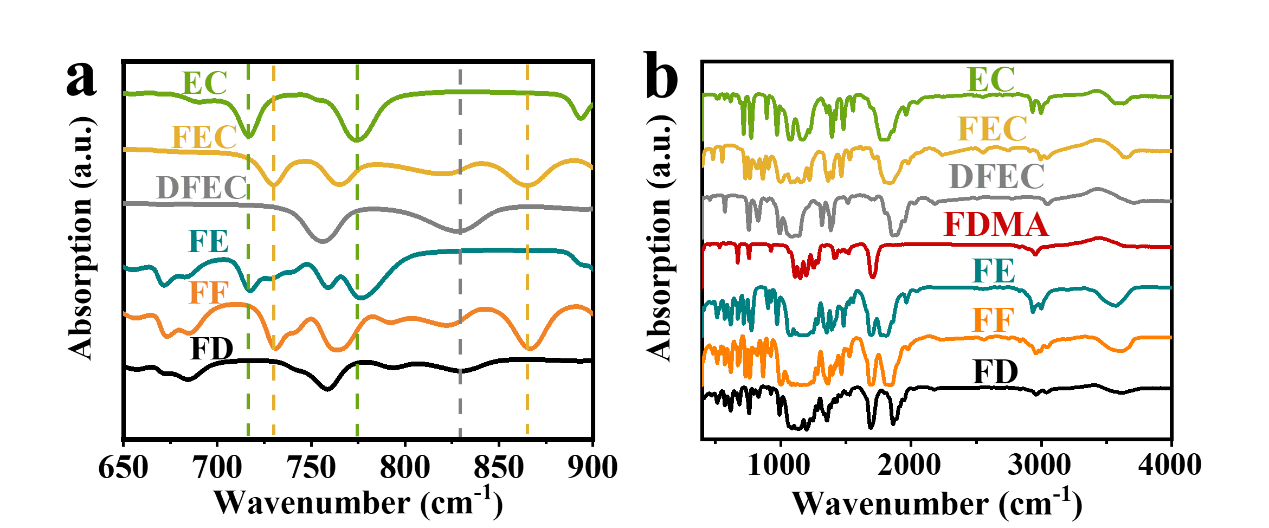


**Figure S2**. FTIR spectra of various fluorinated electrolytes, EC, FEC, DFEC and FDMA at a) 650-900 cm^-1^ and b) 400-4000 cm^-1^.


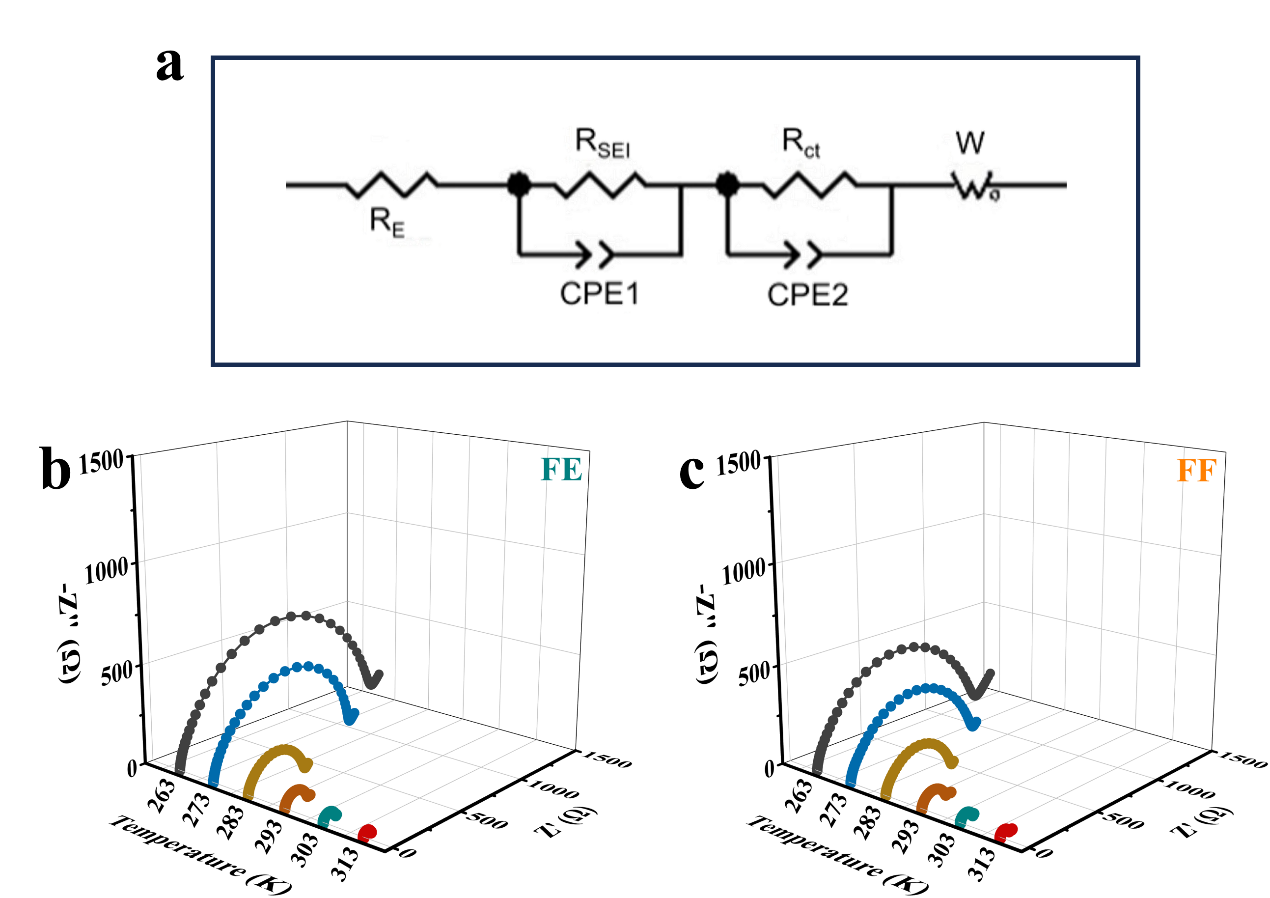


**Figure S3**. Diagram of the fitting equivalent circuit. Nyquist plots of Li||Li cells with b) FE and c) FF electrolytes at temperatures from 263 K to 313 K.


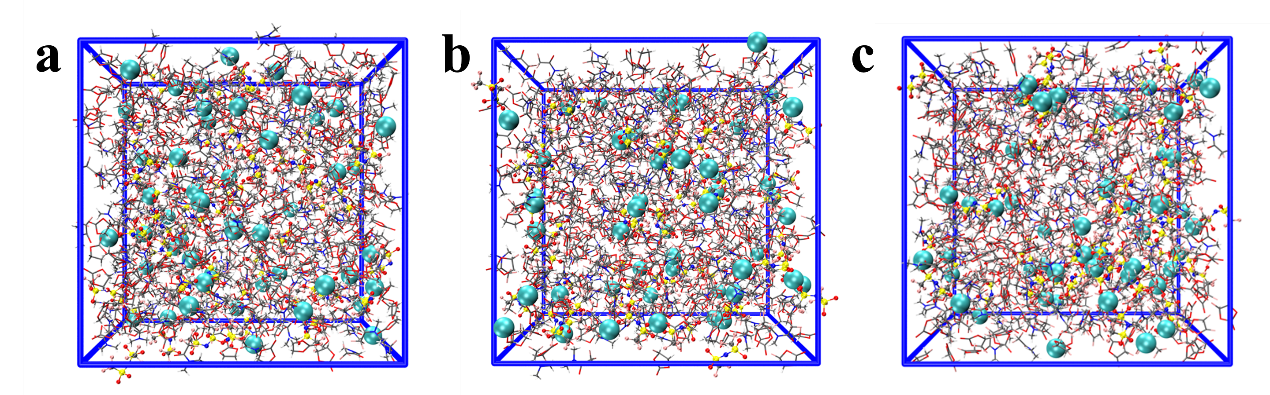


**Figure S4**. Snapshots of a) FE, b) FF, and c) FD electrolytes given in the MD simulation boxes.


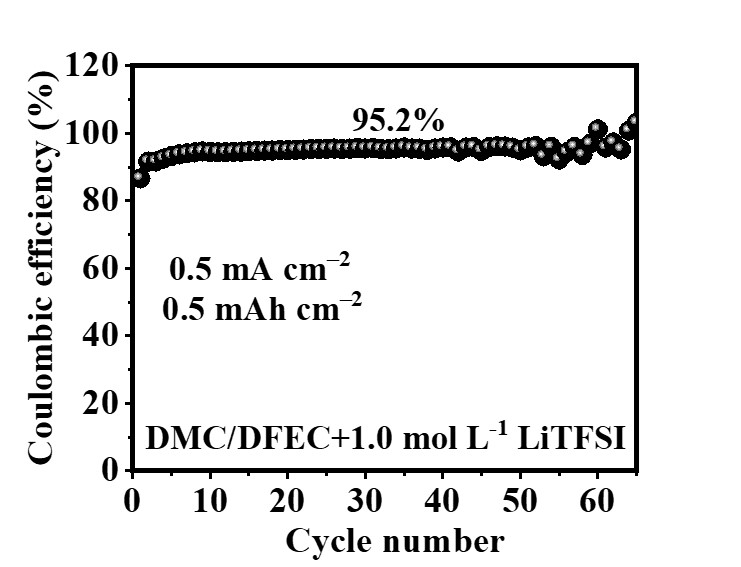


**Figure S5**. Li depositing/stripping CE in Li||Cu cell at 0.5 mA cm^−2^ with a capacity of 0.5 mAh cm^−2^ in DMC/DFEC+1.0 mol L^-1^ LiTFSI electrolyte.

**
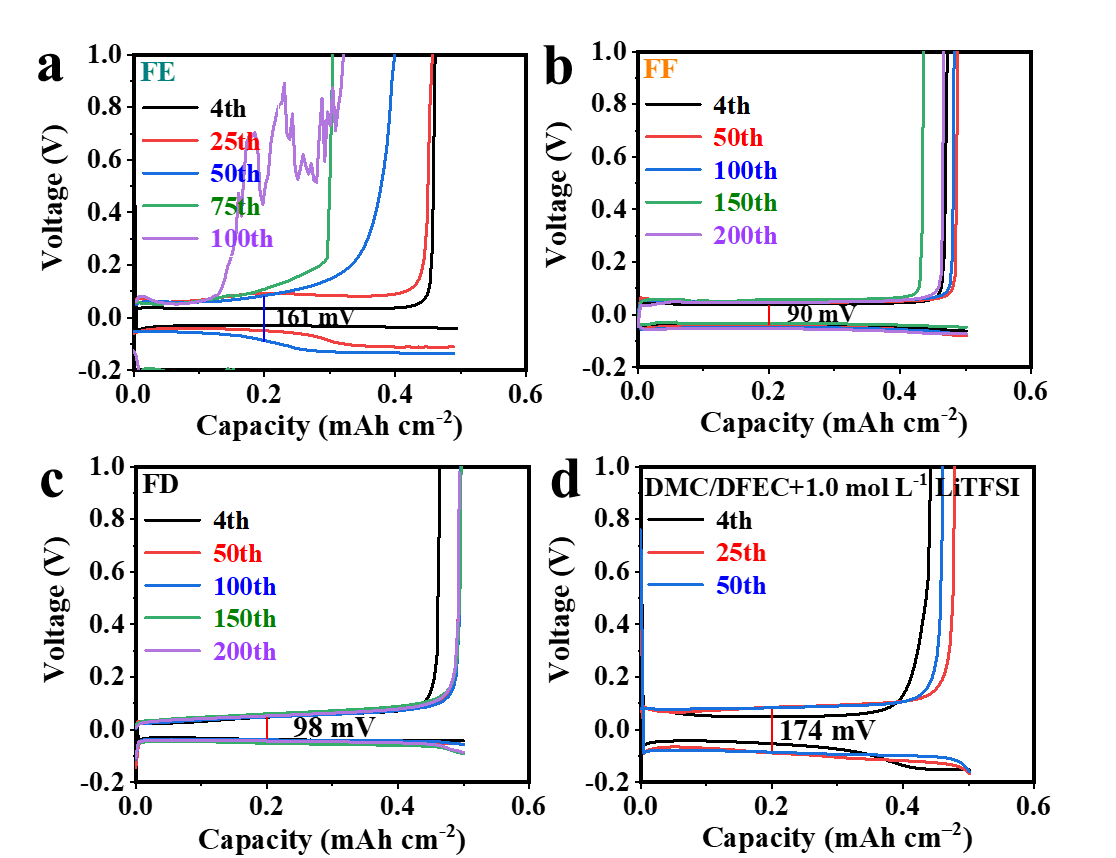
**

**Figure S6**. The voltage curves of Li||Cu cells in a) FE, b) FF, c) FD, and d) DMC/DFEC+1.0 mol L^-1^ LiTFSI electrolytes under the current density of 0.5 mA cm^-2^.


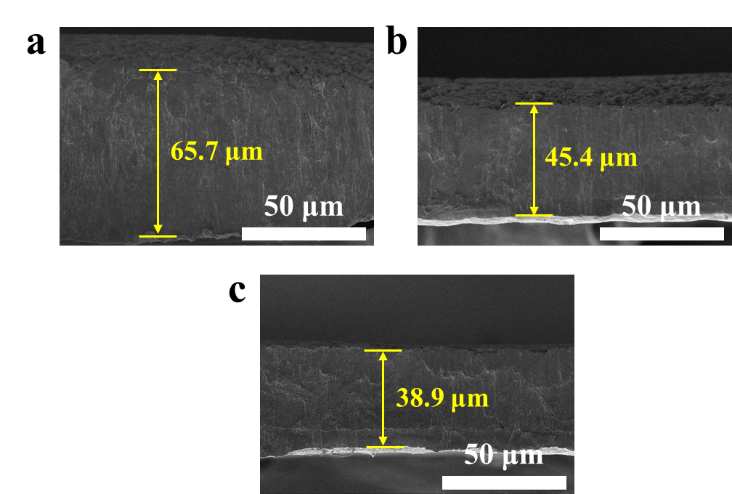


**Figure S7**. The SEM cross-sections morphologies of Li deposition on Cu substrates in a) FE, b) FF, and c) FD electrolytes at 0.5 mA cm^–2^ and 5 mAh cm^–2^.

**Figure S8**. The ionic conductivities of 1 mol L^-1^ LiTFSI dissolved in EC, FEC, DFEC solvents and FE, FF, FD electrolytes at room temperature.


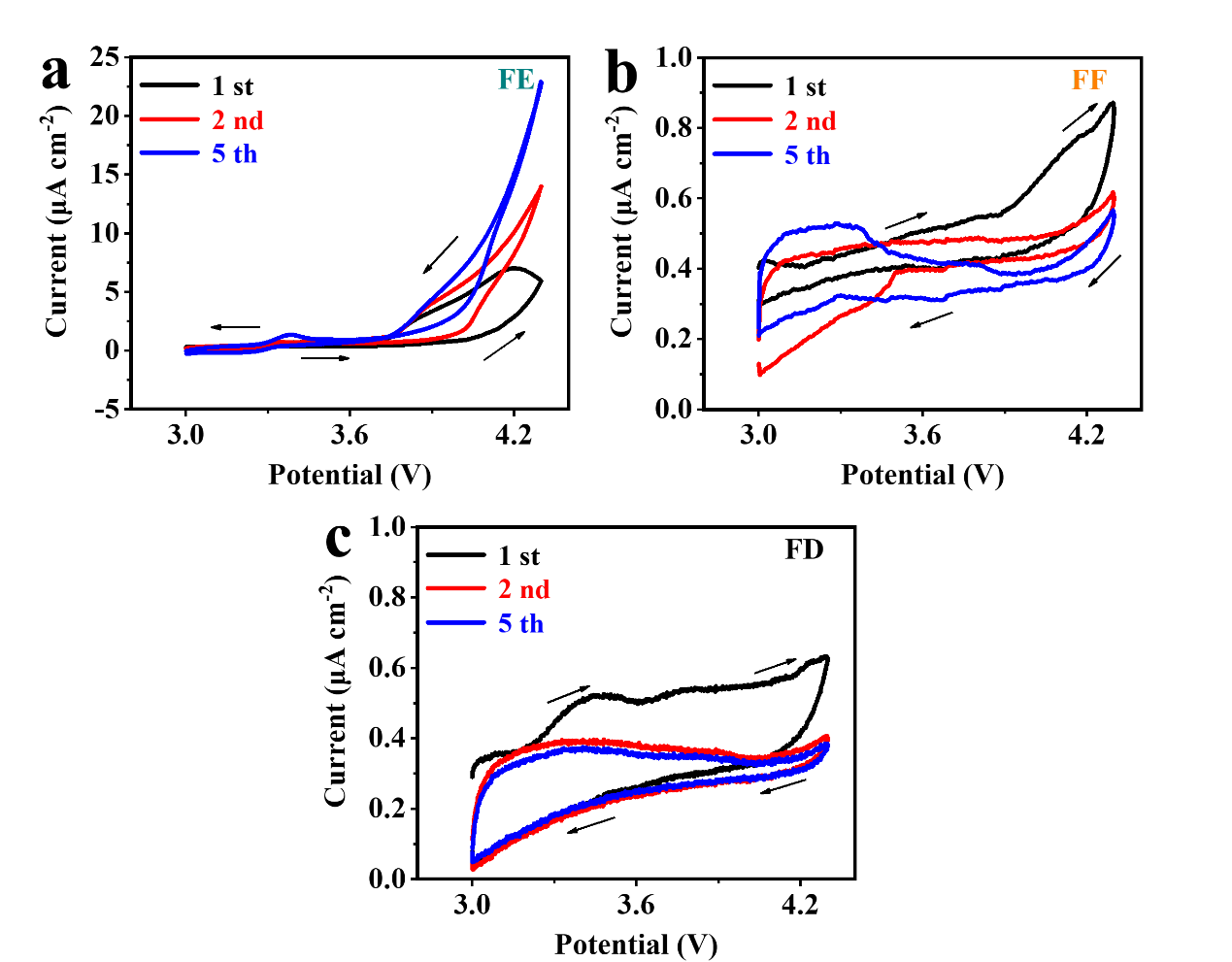


**Figure S9**. CV curves of Li||Al cells using a) FE, b) FF, and c) FD electrolytes for the 1st, 2nd, and 5th cycles at a scan rate of 0.05 mV s^-1^ over the voltage range of 3.0 V-4.3 V.


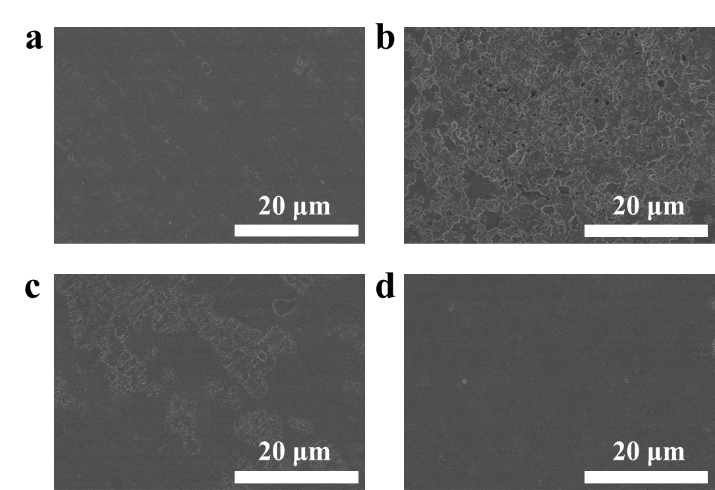


**Figure S10**. SEM images of the Al surface after 5 cycles of CV in the voltage range of 3.0 V-4.3 V at a scan rate of 0.05 mV s^-1^ for a) pure Al foils and Li||Al cells using b) FE, c) FF and d) FD electrolytes.


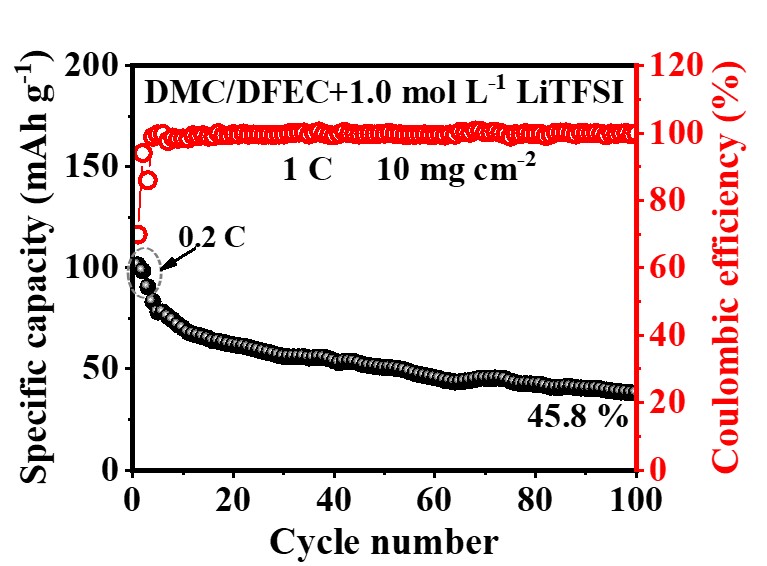


**Figure S11**. Cycling and CE of Li||NCM622 coin cell in DMC/DFEC+1.0 mol L^-1^ LiTFSI electrolyte.


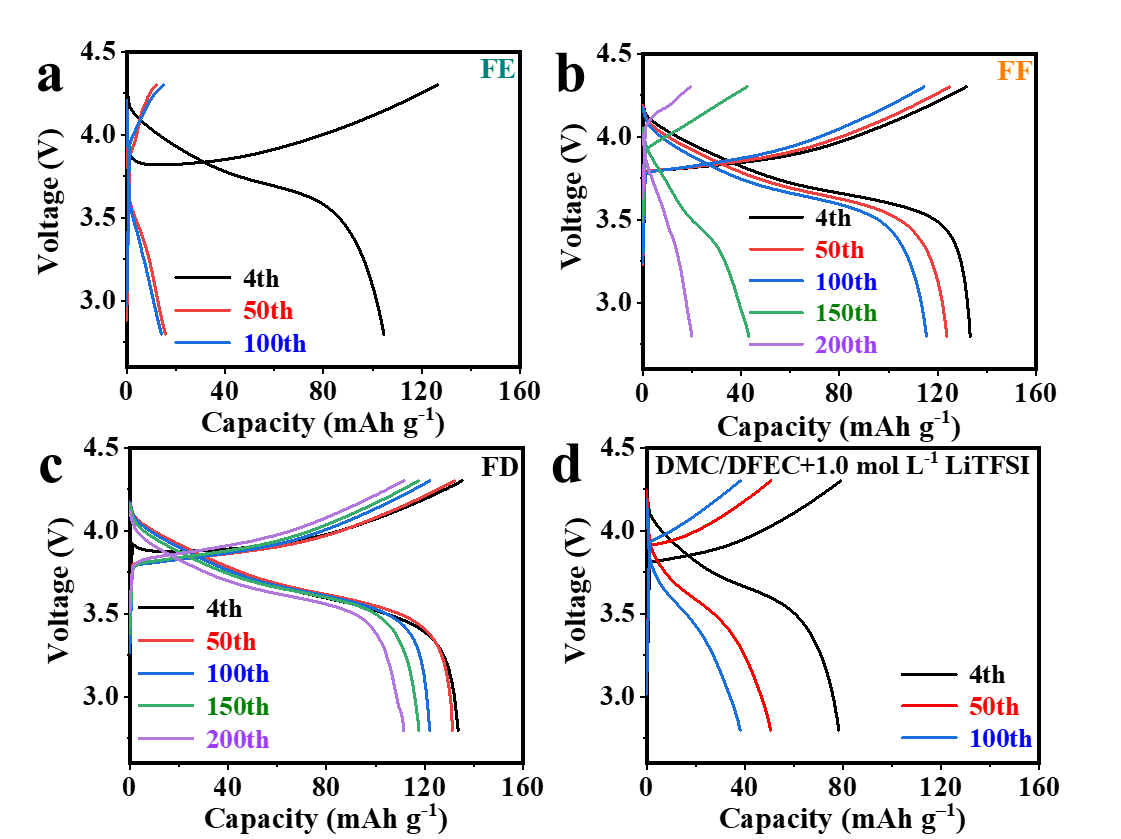


**Figure S12**. Electrochemical charge/discharge curves of the Li||NCM622 coin cells in a) FE, b) FF, c) FD, and d) DMC/DFEC+1.0 mol L^-1^ LiTFSI electrolytes between 2.8 V and 4.3 V.


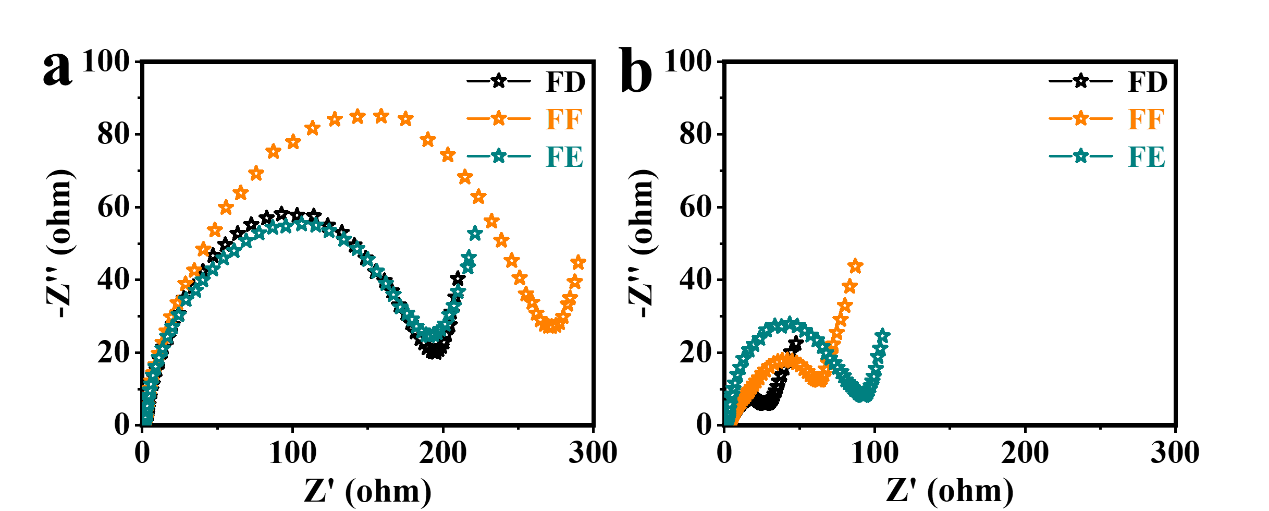


**Figure S13**. Nyquist plots of Li||NCM622 coin cells in various fluorinated electrolytes a) before and b) after 20 cycles at 1 C.


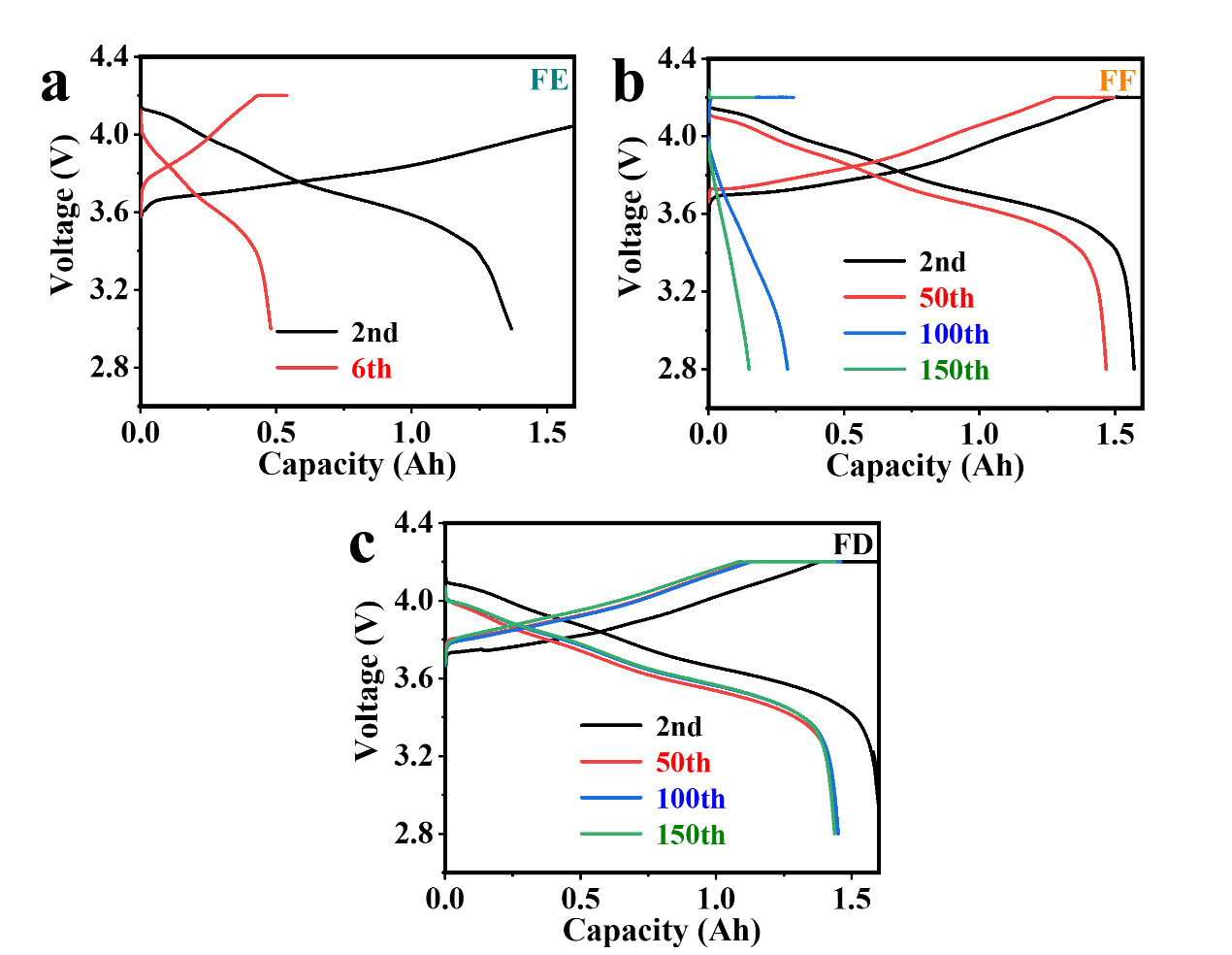


**Figure S14**. Electrochemical charge/discharge curves of the Li||NCM811 pouch cells in a) FE, b) FF and c) FD electrolytes between 2.8 V and 4.2 V.

**Figure S15**. 3D visual images of C_2_HO^-^ fragments when using FE, FF and FD electrolytes.


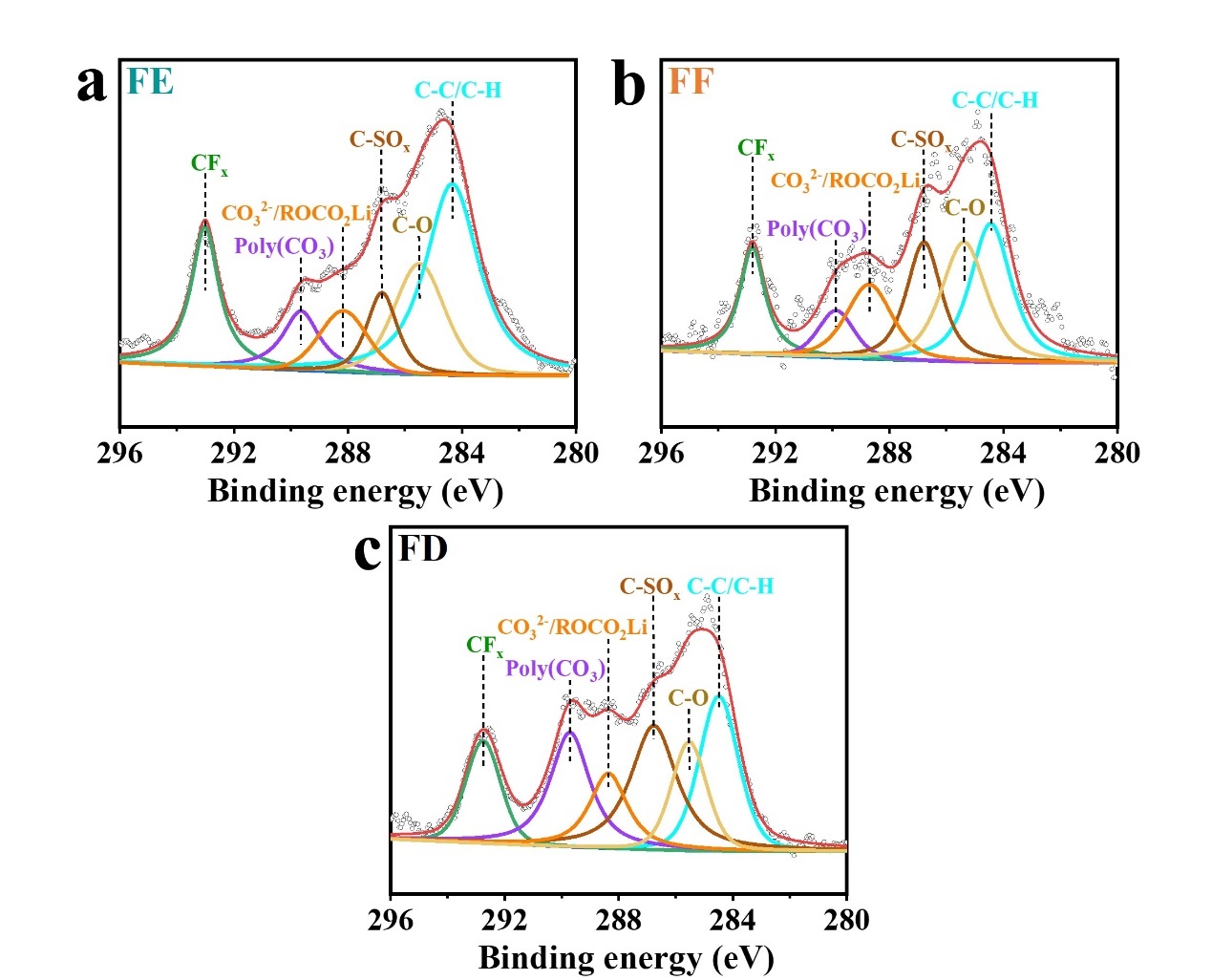


**Figure S16**. XPS spectra of C1s regions of Li anode surfaces collected from the Li||NCM622 coin cells after 30 cycles in a) FE, b) FF and c) FD electrolytes.


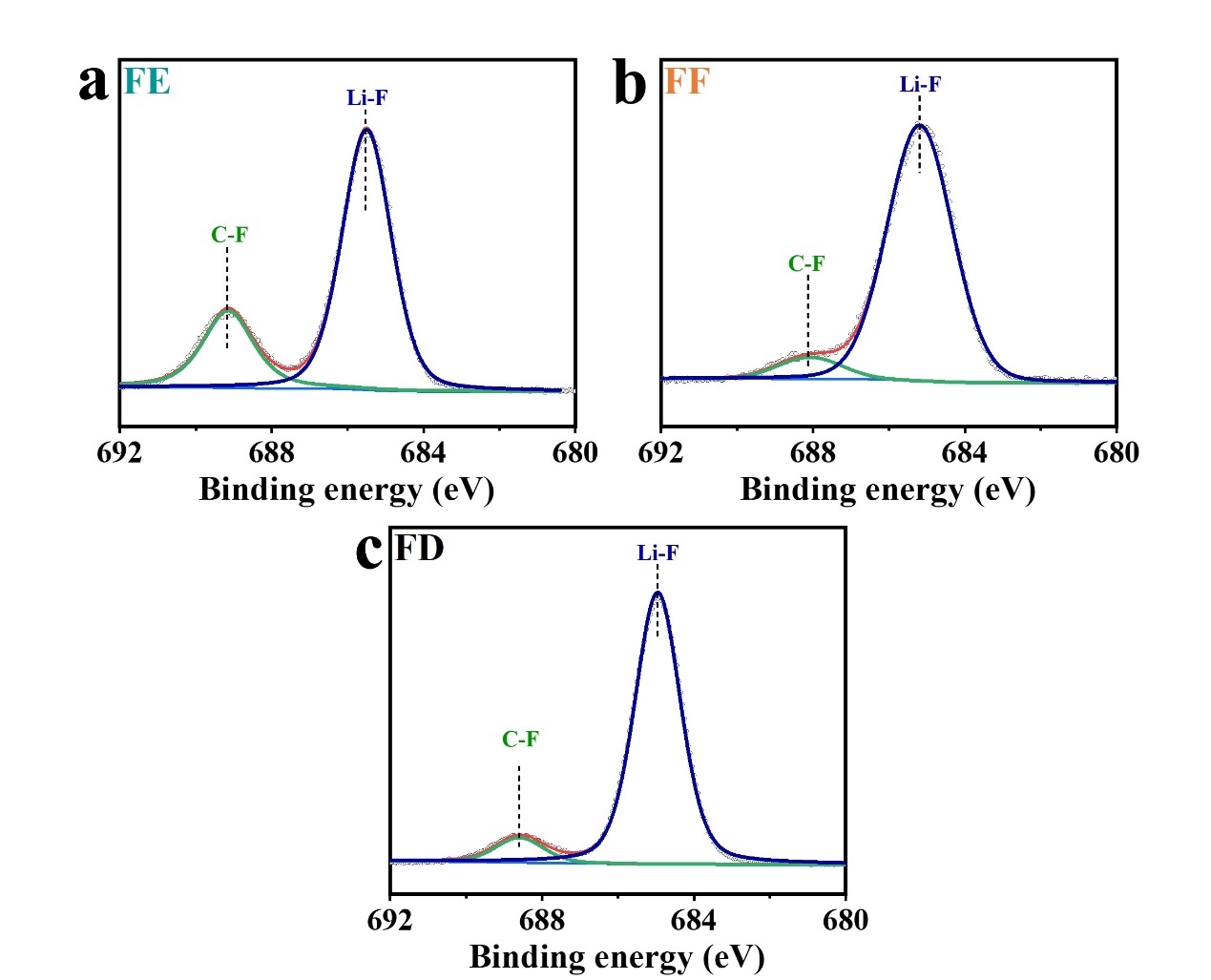


**Figure S17**. XPS spectra of F1s regions of Li anode surfaces collected from the Li||NCM622 coin cells after 30 cycles in a) FE, b) FF and c) FD electrolytes.


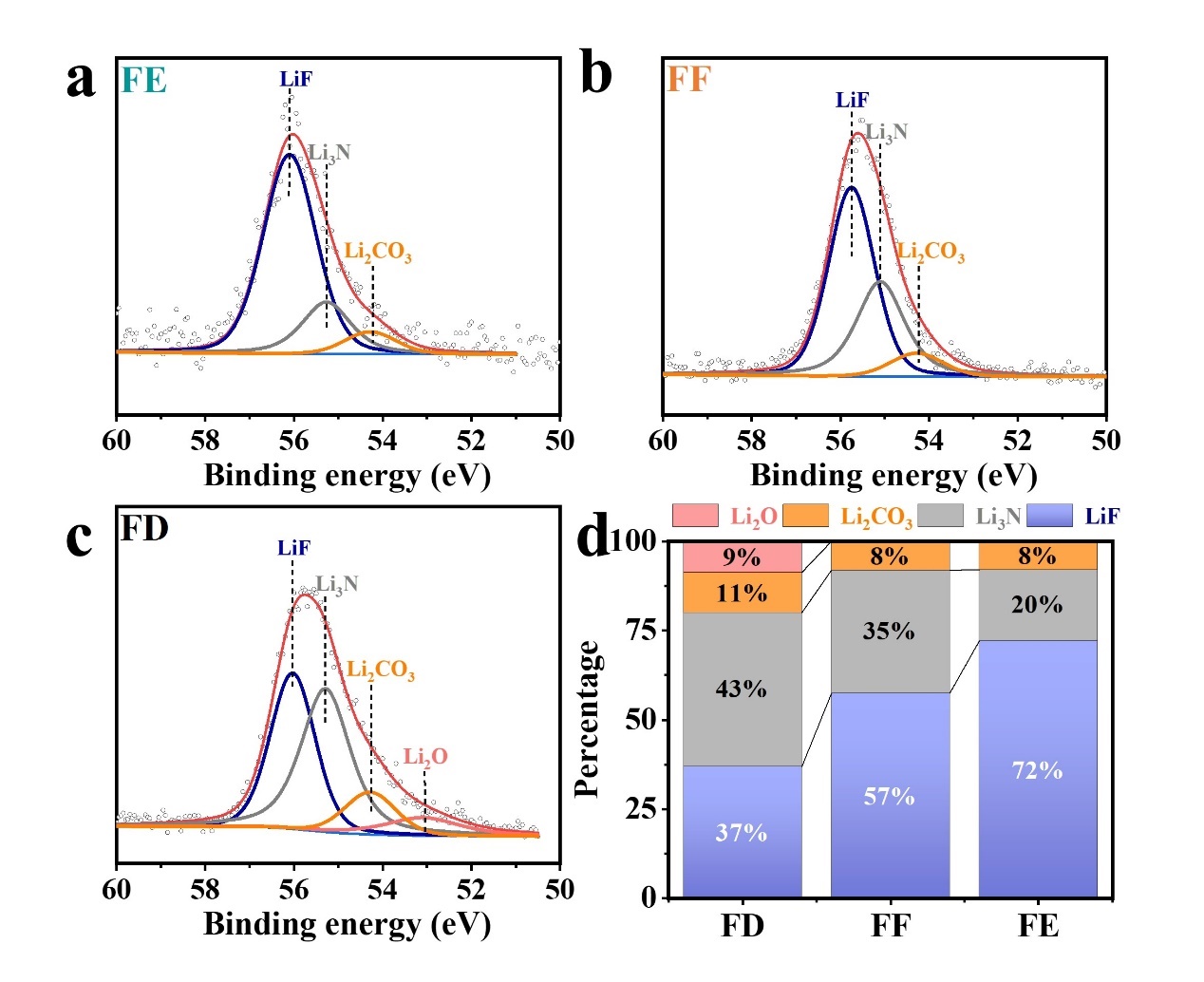


**Figure S18**. XPS spectra of Li1s regions of Li anode surfaces collected from the Li||NCM622 coin cells after 30 cycles in a) FE, b) FF and c) FD electrolytes. d) The relative composition of Li-containing species.

**Figure S19**. XPS spectrum of F1s region of Li anode surface collected from the Li||Li symmetric cell after 30 cycles in 1.0 mol L^-1^ LiClO_4_/FDMA electrolyte.


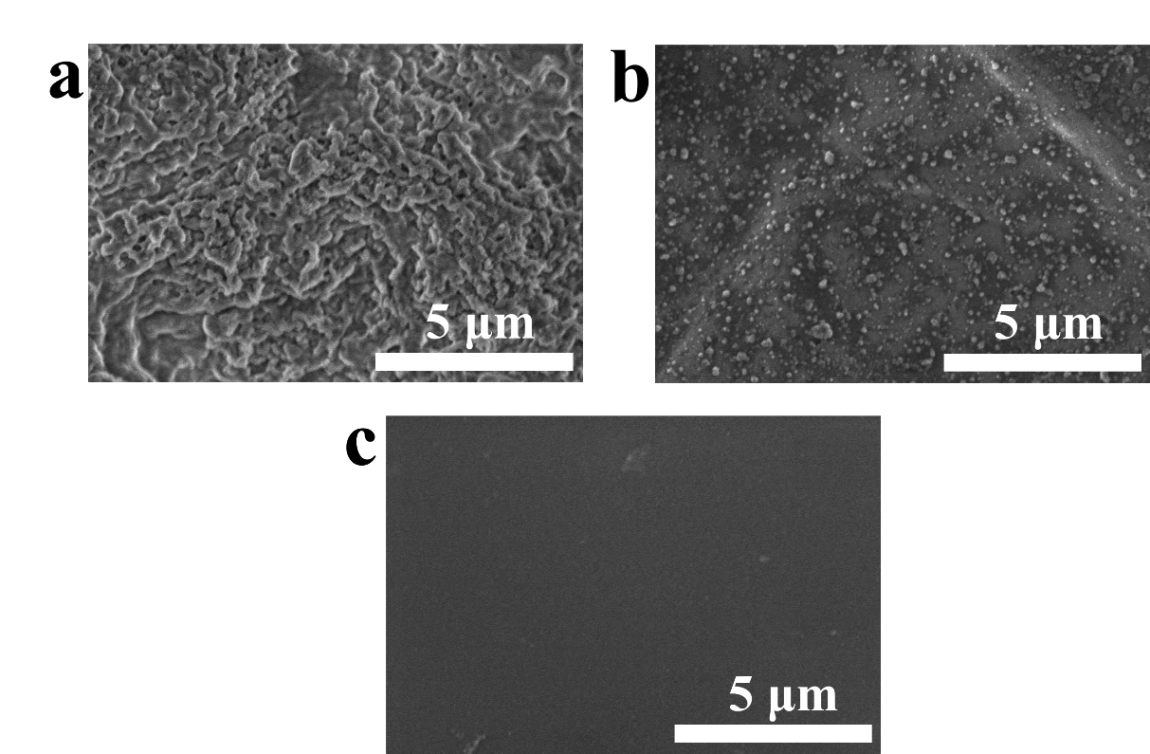


**Figure S20**. SEM images of Li anodes collected from the Li||NCM622 coin cells after 30 cycles in a) FE, b) FF and c) FD electrolytes.


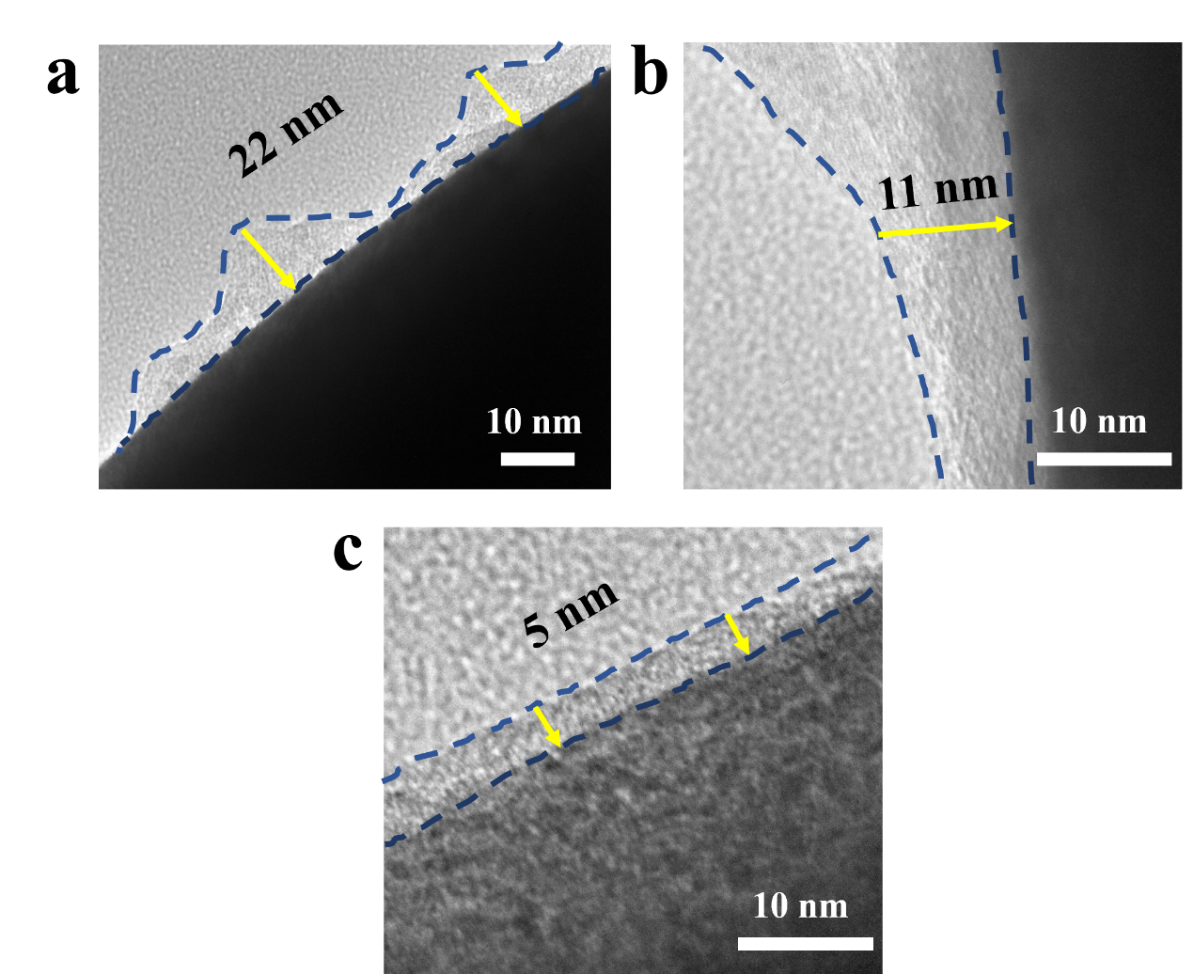


**Figure S21**. TEM images of NCM622 cathodes collected from the Li||NCM622 coin cells after 30 cycles in a) FE, b) FF and c) FD electrolytes.


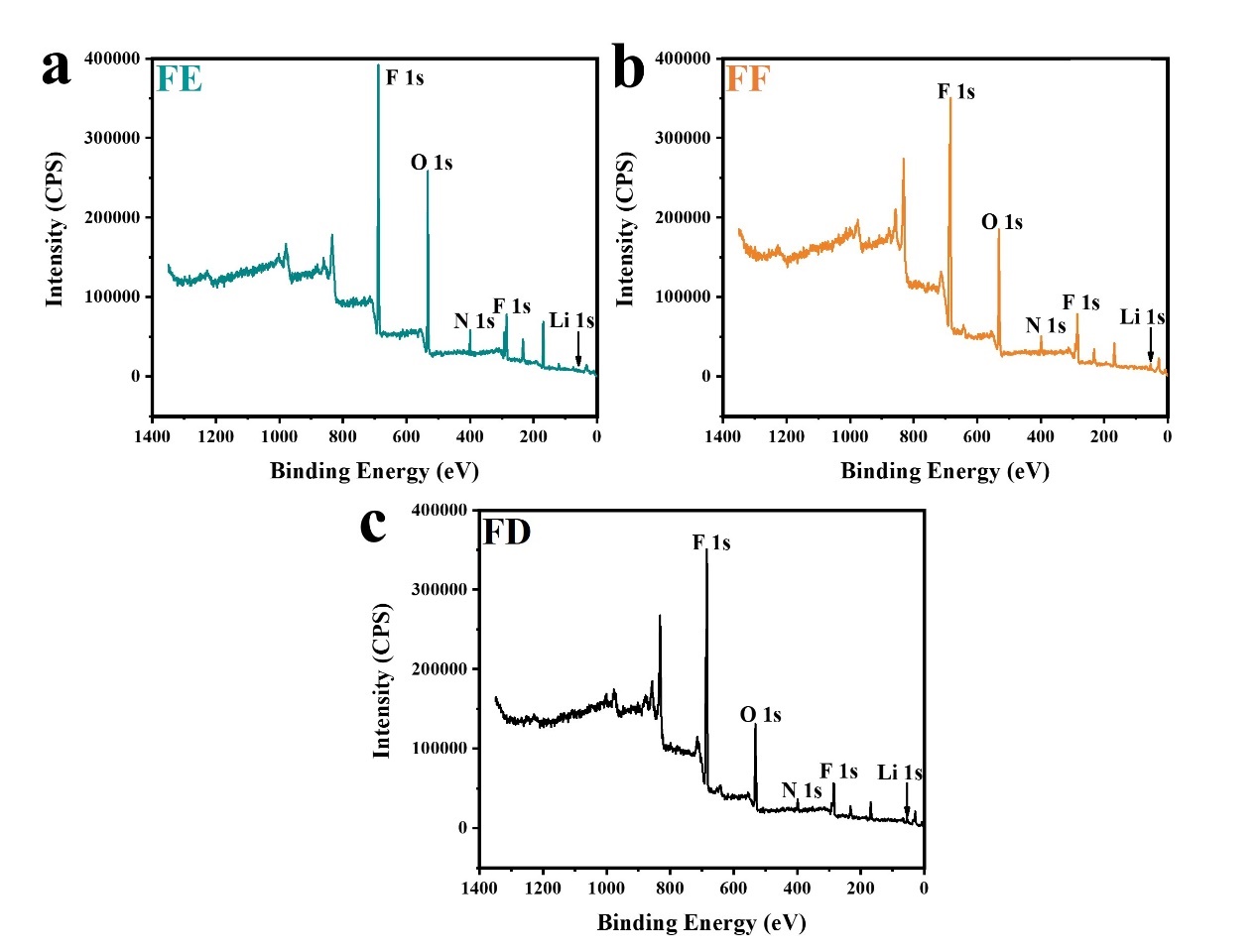


**Figure S22**. XPS spectra of NCM622 cathodes collected from the Li||NCM622 coin cells after 30 cycles in a) FE, b) FF and c) FD electrolytes.


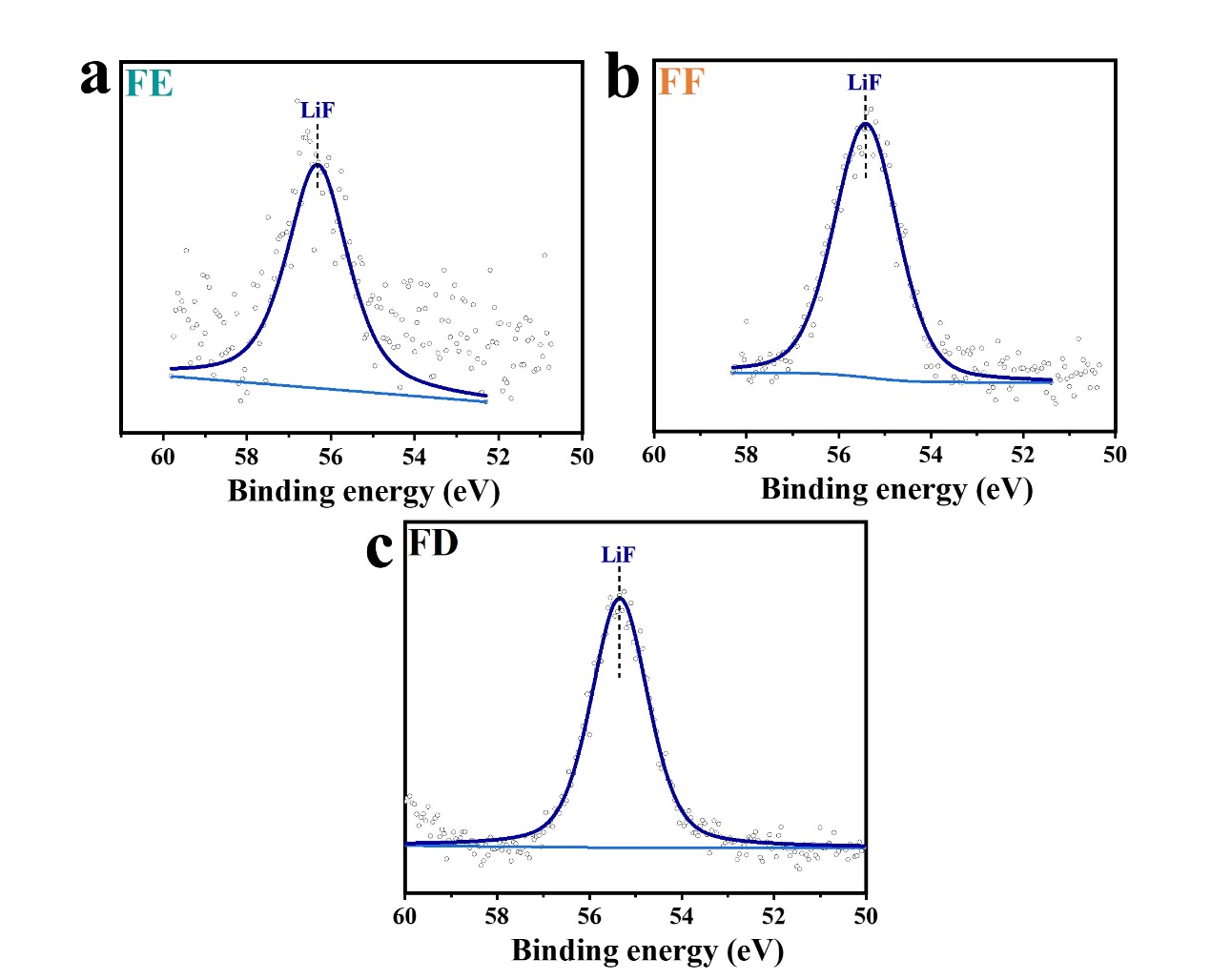


**Figure S23**. XPS spectra of Li1s regions of NCM622 cathodes collected from the Li||NCM622 coin cells after 30 cycles in a) FE, b) FF and c) FD electrolytes.


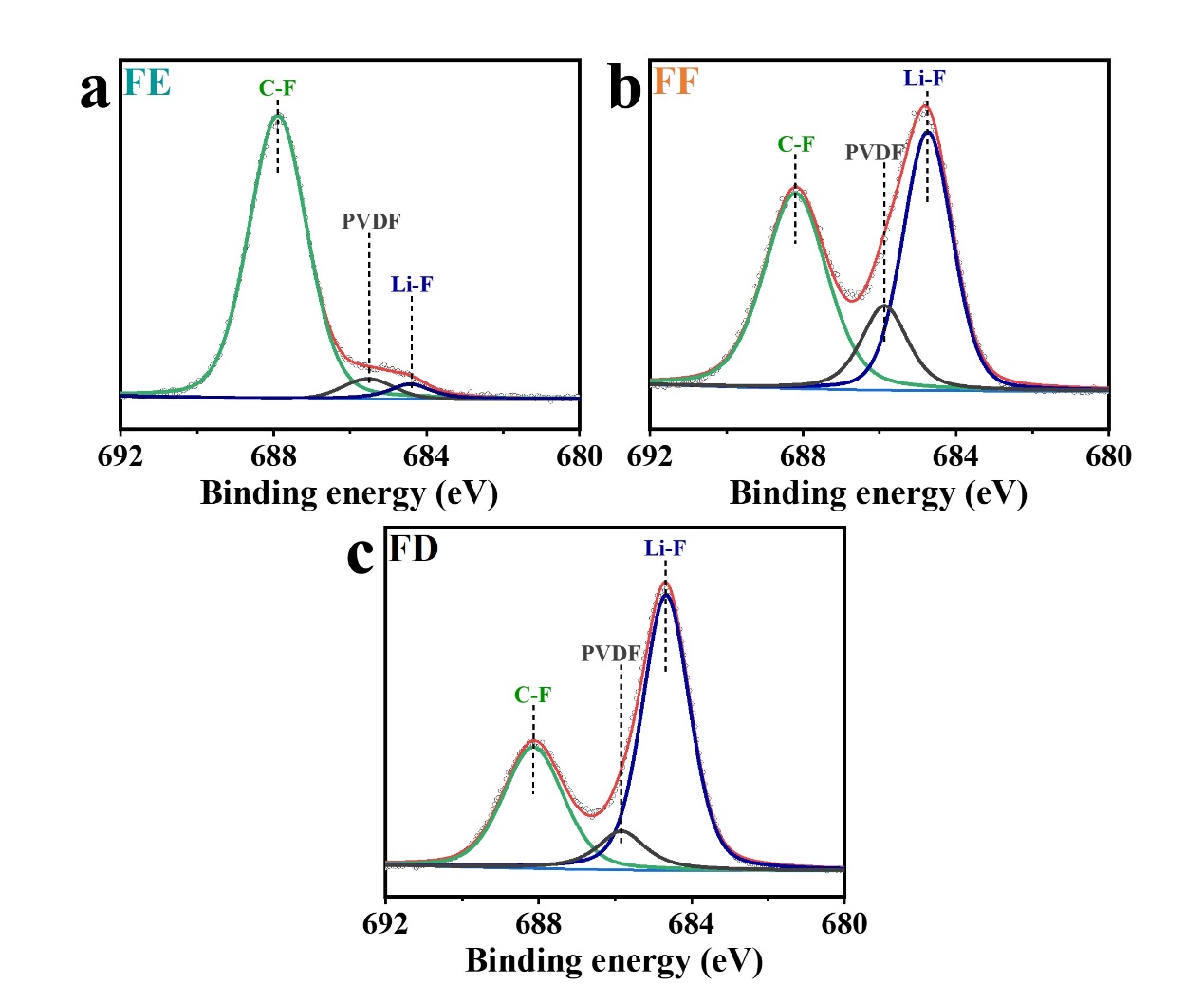


**Figure S24**. XPS spectra of F1s regions of NCM622 cathodes collected from the Li||NCM622 coin cells after 30 cycles in a) FE, b) FF and c) FD electrolytes.


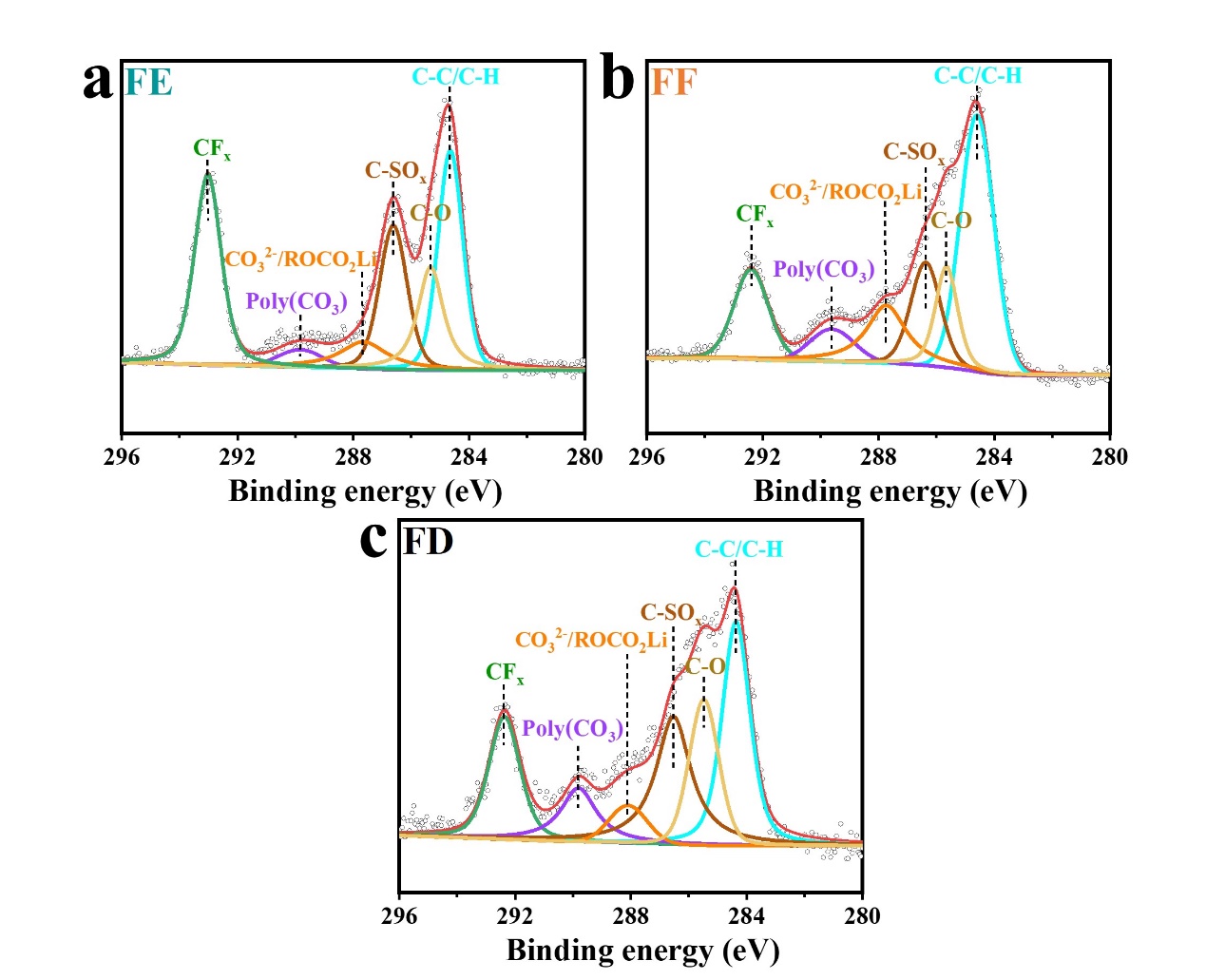


**Figure S25**. XPS spectra of C1s regions of NCM622 cathodes collected from the Li||NCM622 coin cells after 30 cycles in a) FE, b) FF and c) FD electrolytes.

**Table S1.** The distances of Li^+^ with TFSI^-^ anions/solvent molecules in the primary solvation sheaths in different fluorinated electrolytes, which were obtained by RDF analysis. (Unit: Å)

|  | Li^+^-O (TFSI^-^) | Li^+^-O (FDMA) | Li^+^-O (EC) | Li^+^-O (FEC) | Li^+^-O (DFEC) |
| --- | --- | --- | --- | --- | --- |
| FE | 2.04 | 2.08 | 2.10 | - | - |
| FF | 2.02 | 2.06 | - | 2.12 | - |
| FD | 2.02 | 2.06 | - | - | 2.20 |

**Table S2.** The calculated coordination numbers of the Li^+^ with TFSI^-^/solvent molecules.

|  | TFSI^-^ | FDMA | EC | FEC | DFEC | Total* |
| --- | --- | --- | --- | --- | --- | --- |
| FE | 1.242 | 1.780 | 2.562 | - | - | 4.342 |
| FF | 1.449 | 2.105 | - | 1.855 | - | 3.960 |
| FD | 1.890 | 2.674 | - | - | 0.563 | 3.237 |

*****Total=solvent1+solvent2; solvent1=FDMA, solvent2=EC, FEC or DFEC.

**Table S3.** The calculated HOMO/LUMO energy levels of the pure solvents and various complexes in various fluorinated electrolytes.

| Solvent HOMO (eV) LUMO (eV) Energy gap (eV) | | | |
| --- | --- | --- | --- |
| FDMA | -7.32 | -0.47 | 6.85 |
| EC | -8.22 | 0.63 | 8.85 |
| FEC | -8.70 | 0.31 | 9.01 |
| DFEC | -9.20 | -0.21 | 8.99 |
| Li^+^-FDMA/EC | -11.24 | -4.48 | 6.76 |
| Li^+^-FDMA/FEC | -11.30 | -4.56 | 6.74 |
| Li^+^-FDMA/DFEC | -11.39 | -4.63 | 6.76 |
| FDMA/EC-TFSI^-^ | -4.85 | 1.85 | 6.70 |
| FDMA/FEC-TFSI^-^ | -4.89 | 1.72 | 6.61 |
| FDMA/DFEC-TFSI^-^ | -4.75 | 1.87 | 6.62 |
| Li^+^-FDMA/EC-TFSI^-^ | -7.79 | -0.97 | 6.72 |
| Li^+^-FDMA/FEC-TFSI^-^ | -7.89 | -1.33 | 6.73 |
| Li^+^-FDMA/DFEC-TFSI^-^ | -7.86 | -1.22 | 6.64 |

**Table S4.** The DN values of the solvents.

| FDMA^[1]^ | EC^[2]^ | FEC^[3]^ | DFEC |
| --- | --- | --- | --- |
| 22.1 | 16.4 | 7.9 | 2.1 |

**Table S5.** Comparisons of electrochemical performances of the reported electrolytes using in LMBs.

| Electrolyte | Cathode (mg cm^-2^) | Li metal thickness (μm) | Electrolyte amount (μL) | Rate (C) | Cycle number | Retention (%) | Reference |
| --- | --- | --- | --- | --- | --- | --- | --- |
| 1 mol L^-1^ LiTFSI in FDMA/DFEC (1:1 by volume) | NCM622 (10.0) | 50 | 20 | 1.00 | 200 | ~83.7 | This work |
| 1.7 mol L^-1^ LiFSI in DME/TTE (2:7 by volume) | NCM811 (15.3) | 50 | 12 | 0.33 | 155 | ~80.0 | [4] |
| 0.3 mol L^-1^ LiDFOB+0.2 mol L^-1^ LiBF_4_ in DEC/FEC/FB (3.5:1.5:5.0 by volume) | LCO (20.4) | 50 | 17 | 0.25 | 120 | ~85.6 | [5] |
| 1.0 mol L^-1^ LiPF_6_ in FEC/FDEC (3:7 by volume) | NCM811 (8.0) | 50 | 30 | 0.50 | 100 | ~85.7 | [6] |
| 1.0 mol L^-1^ LiPF_6_ in FEC/EMC+1 wt% TPFPB +3 wt% LiNO_3_ | LCO (10.0) | 46 | 11 | 1.00 | 140 | ~80.0 | [7] |
| 1.0 mol L^-1^ LiPF6 in EC/DEC+ 0.3 wt% TAEC | LCO (4.0) | ＞200 | - | 1.00 | 100 | ~85.1 | [8] |
| 1.0 mol L^-1^ LiPF_6_ in EC/DMC+2% TTSPi+0.5%FDEC | LCP (4.1) | ＞200 | 120 | 1.00 | 80 | ~85.0 | [9] |
| 1.0 mol L^-1^ LiPF_6_ in EC/DMC+0.1 wt% DEPP | LCO (2.0) | ＞200 | - | 1.00 | 100 | ~69.5 | [10] |
| 1.0 mol L^-1^ LiFSI in DME/TEEO (1.2:3 by mol) | NCM811 (7.5) | 50 | 75 | 0.33 | 200 | ~80.0 | [11] |
| 0.95 mol L^-1^ LiN(SO_2_F)_2_ in TFEP/FEMC (1:3 by volume) | LNMO (4.0) | 400 | - | 0.50 | 200 | ~70.0 | [12] |
| 1.0 mol L^-1^ LiFSI in DTDL | NCM811 (5.0) | 20 | 40 | 0.50 | 200 | ~84.0 | [13] |
| 1.0 mol L^-1^ LiTFSI in SL/HFE/ FEC (1:1:1 by volume) | NCM811 (3.0) | 400 | 50 | 0.50 | 150 | ~85.0 | [14] |
| 1.0 mol L^-1^ LiPF_6_ in EC/FPES (1:1 by weight) | NCM622 (2.0) | ＞200 | - | 0.05 | 100 | ~73.0 | [15] |
| LiFSI/AN/VC (0.52:1:0.09 by mol) | NCM622 (21.6) | 500 | 70 | 0.50 | 200 | ~59.8 | [16] |

**Table S6.** Cell parameter of 5.9 Ah Li||NCM811 pouch cell.

| Cell Component Parameter Value | | |
| --- | --- | --- |
| NMC811 cathode | discharge capacity (mAh g^-1^) | 200 |
|  | active material loading | 97.5% |
|  | total coating weight (mg cm^-2^ each side) | 20 |
|  | areal capacity (mAh cm^-2^ each side) | 3.8 |
|  | electrode press density (g cm^-3^) | 3.4 |
|  | electrode length (mm) | 80 |
|  | electrode width (mm) | 64 |
|  | electrode thickness (single side) (μm) | 71 |
|  | Al foil thickness (μm) | 12 |
|  | layers | 14 |
| Li anode electrode thickness (μm) 50 | | |
| Electrolyte electrolyte/capacity (g Ah^-1^) 2 | | |
| Separator thickness (μm) 9 | | |
| Packaging foil thickness (μm) 76 | | |
| Cell | voltage (V) | 3.8 |
|  | capacity (Ah) | 5.9 |
|  | weight (g) | 50.1 |
|  | energy density (Wh kg^-1^) | 447 |

**References**

[1] F. B. Zeng, S. L. Li, S. L. Hu, M. L. Qiu, G. B. Zhang, M.L. Li, C. Y. Chang, H. L. Wang, M. W. Xu, L. R. Zheng, Y. B. Tang, C. P. Han, H. M. Cheng, *Adv. Funct. Mater.* **2024**, *34*, 2302397.

[2] Y. Z. Wu, Q. Hu, H. M. Liang, A. P. Wang, H. Xu, L. Wang, *Adv. Energy Mater*. **2023**, *13*, 2300259.

[3] Y. H. Liang, C. Shen, H. Liu, C. Wang, D. B. Li, X. X. Zhao, L.-Z. Fan, *Adv. Sci*. **2023**, *10*, 2300985.

[4] X. D. Ren, L. F. Zhou, X. Cao, M. H. Engelhard, W. Liu, S. D. Burton, H. Lee, C. J. Niu, B. E. Matthews, Z. H. Zhu, C. M. Wang, B. W. Arey, J. Xiao, J. Liu, J.-G. Zhang, W. Xu, *Joule* **2019**, *3*, 1662.

[5] Z. P. Jiang, Z. Q. Zeng, H. Zhang, L. Yang, W. Hu, X. M. Liang, J. W. Feng, C. Yu, S. J. Cheng, J. Xie, *iScience*, **2022**, *25*, 103490.

[6] P. T. Xiao, Y. Zhao, Z. H. Piao, B. Li, G. Zhou, H. M. Cheng, *Energy Environ. Sci*. **2022**, *15*, 2435.

[7] S. Y. Li, W. D. Zhang, Q. Wu, L. Fan, X. Y. Wang, X. Wang, Z. Y. Shen, Y. He, Y. Y. Lu, *Angew. Chem., Int. Ed*. **2020**, *59*, 14935.

[8] Y. Zheng, W. Fang, H. Zheng, Y. Su, X. Liang, C. H. Chen, H. F. Xiang, *J. Electrochem. Soc*. **2019**, *166*, 3222.

[9] A. Kazzazi, D. Bresser, M. Kuenzel, M. Hekmatfar, J. Schnaidt, Z. Jusys, T. Diemant, R. J. Behm, M. Copley, K. Maranski, J. Cookson, I. D. Meatza, P. Axmann, M. W.- Mehrens, S. Passerini, *J. Power Sources* **2021**, *482*, 228975.

[10] C. X. Miao, S. H. Qi, K. Liang, Y. L. Qi, J. D. Huang, M. G. Wu, H. S. Zhao, J. D. Liu, Y. R. Ren, J. M. Ma, *J. Energy Chem*. **2021**, *63*, 566.

[11] X. Cao, L. F. Zou, B. E. Matthews, L. C. Zhang, X. Z. He, X. D. Ren, M. H. Engelhard, S. D. Burton, P. Z. E.- Khoury, H.-S. Lim, C. J. Niu, H. Lee, C. S. Wang, B. W. Arey, C. M. Wang, J. Xiao, J. Liu, W. Xu, J.-G. Zhang, *Energy Storage Mater*. **2021**, *34*, 76.

[12] Q. F. Zheng, Y. K. Yamada, R. Shang, S. Ko, Y.-Y. Lee, K. Kim, E. Nakamura, A. Yamada, *Nature Energy* **2020**, *5*, 291.

[13] Y. Zhao, T. H. Zhou, T. Ashirov, M. E. Kazzi, C. Cancellieri, L. P. H. Jeurgens, J. W. Choi, A. Coskun, *Nat. Commun*. **2022**, *13*, 2575.

[14] W. B. Hou, D. L. Zhu, S. D. Ma, W. Yang, H. Yan, Y. Dai, *J. Power Sources* **2022**, *517*, 230683.

[15] D. Steinle, Z. Chen, H. D. Nguyen, M. Kuenzel, C. Iojoiu, S. Passerini, D. Bresser, *J Solid State Electrochem*. **2022**, *26*, 97.

[16] Z. Peng, X. Cao, P. Y. Gao, H. P. Jia, X. D. Ren, S. Roy, Z. D. Li, Y. Zhu, W. P. Xie, D. Y. Liu, Q. Y. Li, D. Y. Wang, W. Xu, J.-G. Zhang, *Adv. Funct. Mater*. **2020**, *30*, 2001285.
